# Biological and Spectroscopic Investigations of New Tenoxicam and 1.10-Phenthroline Metal Complexes

**DOI:** 10.3390/molecules25051027

**Published:** 2020-02-25

**Authors:** Hazem S. Elshafie, Sadeek A. Sadeek, Ippolito Camele, Amira A. Mohamed

**Affiliations:** 1 School of Agricultural, Forestry, Food and Environmental Sciences, University of Basilicata, Viale dell’Ateneo Lucano 10, 85100 Potenza, Italy; hazem.elshafie@unibas.it; 2 Department of Chemistry, Faculty of Science, Zagazig University, Zagazig 44519, Egypt; s_sadeek@zu.edu.eg; 3 Department of Basic Science, Zagazig Higher Institute of Engineering and Technology, Zagazig 44519, Egypt; amiraabdeldayem6@gmail.com

**Keywords:** H_2_Ten, rheumatoid drug, Phen, thermal analysis, phytotoxicity, MTT cytotoxicity assay

## Abstract

In the present work, tenoxicam (H_2_Ten) reacted with Mn(II), Co(II), Ni(II), Cu(II) and Zn (II) ions in the presence of 1.10-phenthroline (Phen), forming new mixed ligand metal complexes. The properties of the formed complexes were depicted by elemental analyses, infrared, electronic spectra, proton nuclear magnetic resonance (^1^H NMR), mass spectrometry, thermogravimetric (TGA) and differential thermogravimetric (DTG) analysis, molar conductance and magnetic moment. IR spectra demonstrated that H_2_Ten acted as a neutral bidentate ligand, coordinated to the metal ions via the pyridine-N and carbonyl group of the amide moiety, and Phen through the nitrogen atoms. Kinetic thermodynamics parameters activation energy (E*), enthalpy of activation (ΔH*), entropy of activation (ΔS*), Gibbs, free energy (ΔG*) associated to the complexes have been evaluated. Antibacterial screening of the compounds was carried out in vitro against *Clavibacter michiganensis*, *Xanthomonas campestris* and *Bacillus megaterium*. Antifungal activity was performed in vitro against *Monilinia fructicola*, *Penicillium digitatum* and *Colletotrichum acutatum*. The possible phytotoxic effect of the studied compounds was also investigated on *Solanum lycopersicum* (tomatoes) and *Lepidium sativum* (garden cress) seeds. The anticancer activity was screened against cell cultures of HCT-116 (human colorectal carcinoma), HepG2 (human hepatocellular carcinoma) and MCF-7 (human breast adenocarcinoma).

## 1. Introduction

**Scheme 1:**
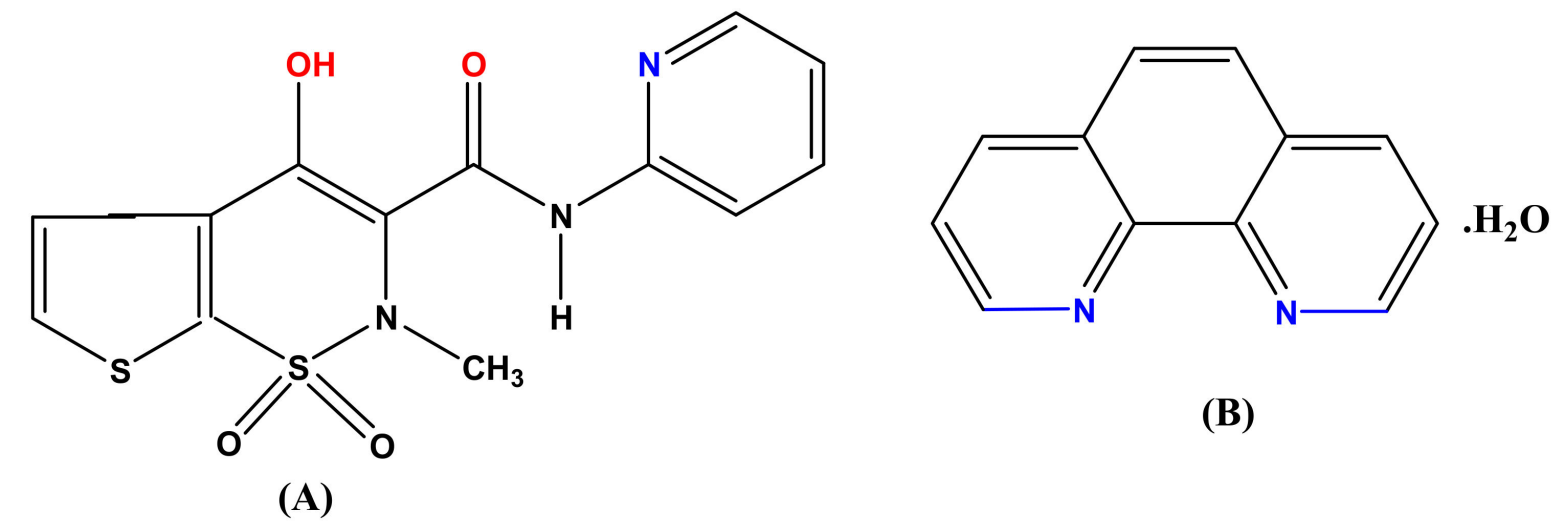
(**A**) H_2_Ten (4-hydroxy-2-methyl-N-(2pyridyl)-2H-thieno [2,3-e]-1,2-thiazine-3-carboxamide 1,1-dioxide) and (**B**) 1,10-phenanthroline monohydrate (Phen).

Recently, non-steroidal anti-inflammatory drugs have been pursued due to their potential applications and biological properties. Presently, non- steroidal anti- inflammatory drugs (NSAIDs) are used as therapeutic treatment [1]. Oxicam is a class of NSAID family, which can be closely attached to plasma proteins and react with metal ions, forming complexes [2]. The formation of oxicam complexes with various metal ions promotes their anti-inflammatory and antimicrobial effects more than free ligands [3,4,5,6]. Tenoxicam (H_2_Ten), (Scheme 1A) is the most studied species of this family and is often used for treating various musculoskeletal and joint disorders, rheumatoid arthritis, knee osteoarthritis, acute gout, and relief of post- surgical inflammation [7,8]. H_2_Ten has a ring of five-membered heteroatoms that innervates the clinical activity of the drug. Generally, H_2_Ten behaves like a bidentate ligand when it is coordinated with the metal ions by the pyridyl ring amide oxygen and nitrogen atom [9,10]. Several studies in human cancer cell lines and animal models, as well as epidemiological and clinical studies, proposed that NSAIDs require precise antineoplastic activity [11,12,13]. Nitrogen based ligands are used in the synthesis and design of compounds for biological, chemotherapeutic, and pharmacological applications, such as anti-rheumatics and anti-histamines [14]. The 1,10-Phenanthroline (Phen) (Scheme 1B) is an efficient chelating nitrogen donor ligand, which produces stable complexes in a solution with metal ions [14] and has additional properties for complexes because it contains heteroaromatic and aromatic groups.

Our research goal was to evaluate the cytotoxicity effect of some new mixed ligand complexes of Mn(II), Co(II), Ni(II), Cu(II), and Zn(II) with H_2_Ten and Phen, against three types of tumor cell lines: HCT-116 (human colorectal carcinoma), HepG2 (human hepatocellular carcinoma), and MCF-7 (human breast adenocarcinoma). Whereas, their antimicrobial activity was evaluated against three phytopathogenic fungi: *Monilinia fructicola* (G. Winter) Honey, *Penicillium digitatum* (Pers.) Sacc., and *Colletotrichum acutatum* J.H. Simmonds, and three bacterial strains: *Clavibacter michiganensis* corrig. (Smith) Davis et al., *Xanthomonas campestris,* and *Bacillus megaterium* (Pammel) Dowson. Phytotoxicity assay was performed against *Solanum lycopersicum* L. (tomatoes) and *Lepidium sativum* L. (cress). The following physical properties were tested for the mixed ligand complexes: elemental analyses, conductivity, magnetic susceptibility measurements in order to establish its molecular formula, infrared spectroscopy (IR), ultra-violet visible spectroscopy (UV–Vis.), proton nuclear magnetic resonance (^1^H NMR), mass spectra, and thermal analyses.

## 2. Results and Discussion

The results of elemental analysis for H_2_Ten and Phen ligands and their complexes (Table S1) were in good agreement with the calculated values, and showed that the reaction of H_2_Ten and Phen with various metal salts in (1:1:1) molar ratio gives complexes with stoichiometric 1:1:1 (H_2_Ten: Phen: M). The complexes are insoluble in common organic solvents, but soluble in dimethyl formamide (DMF) and dimethyl sulfoxide (DMSO). The molar conductance values for (A), (B), (C), (D), and (E) chelates were found to be 172.20, 174.00, 157.39, 171.42, and 176.30 S cm^2^ mol^−1^, respectively. The relatively high values indicate the ionic nature of these chelates and they are of the type 1:1:1 electrolyte [15,16,17]. The magnetic moment values are done and reported in Table 1. (A), (B), (C), (D) complexes have μ_eff_ values 5.44, 5.10, 3.30, and 1.70 B.M, respectively, which assumes a high spin octahedral geometries [16,17,18,19]. On the other hand, (E) complex is diamagnetic and has octahedral geometry structure.

### 2.1. FT-IR Spectra

**Scheme 2:**
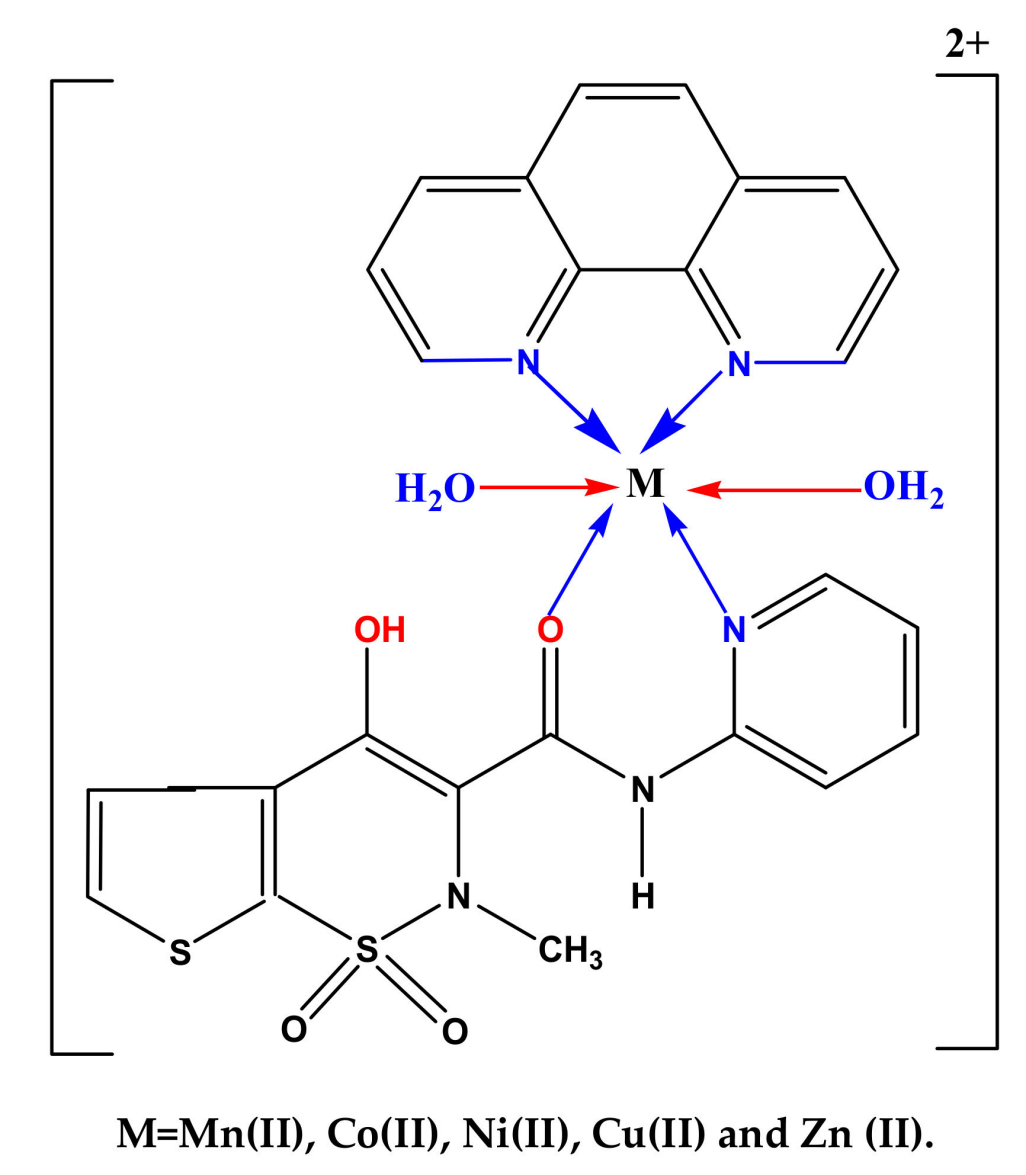
The coordination mode of Mn (II), Co(II), Ni(II), Cu(II), and Zn (II) with H_2_Ten and Phen.

The IR spectra of the free ligands H_2_Ten and Phen and their chelates are indexed in Table 1. The free H_2_Ten spectrum suggested a strong broad band centered at 3432 cm^−1^ function of O-H stretching [20,21,22,23,24]. The amplitude of the O-H band is symptomatic of formation hydrogen bond [25]. The strong band appeared at 1635 cm^−1^, which assigned to the carbonyl stretching vibration of the secondary amide group (–CO–NH–) in the free H_2_Ten (Figure S1). This band is shifted to lower frequency (8–35 cm^−1^) or to higher frequency (5-8 cm^−1^), demonstrating the participation of the C=O of the amide moiety in chelate configuration [16,17,18]. The stretching vibration of ν(C=N) of the pyridyl nitrogen placed at 1599 cm^−1^ is assigned to the coordination of the pyridyl nitrogen is special through an alternate (7–66) cm^−1^ at a lower wave numbers [24,25]. It is found that the vibration of asymmetrical and symmetrical stretching ν(SO_2_) is a strong band that appears at 1327 cm^−1^ and 1040 cm^−1^, respectively, the two SO_2_ bands move at higher or lower frequencies in complexes [26]. Since the SO_2_ group is not implicated in metal bonding, this change at higher or lower frequencies must be correlated to the important hydrogen bonding effects. It has become comprehensible that, in all cases, H_2_Ten acted as a chelate bi dentate ligand via the oxygen atom of the amide group and nitrogen atom of the pyridyl ring (Scheme 2) [18].

The free Phen ring vibration peak was at 1586 cm^−1^ and the Phen peak in the chelates was in the range of 1510–1524 cm^−1^, indicating that the Phen was matched with metal ions by the two atoms of nitrogen and indicated that the Phen had behaved as a bidentate ligand [27]. The bonding is also proven via the presence of new bands of diverse intensity in the IR spectra of the complexes manifestation at 627–423 cm^−1^ assigned to ν(M-O) and ν(M-N) stretching vibrations, respectively [28,29]. It might also be noted that these vibration bands are absent in the infrared spectra of H_2_Ten as well as Phen ligand.

### 2.2. UV–Visible Spectra

**Figure 1:**
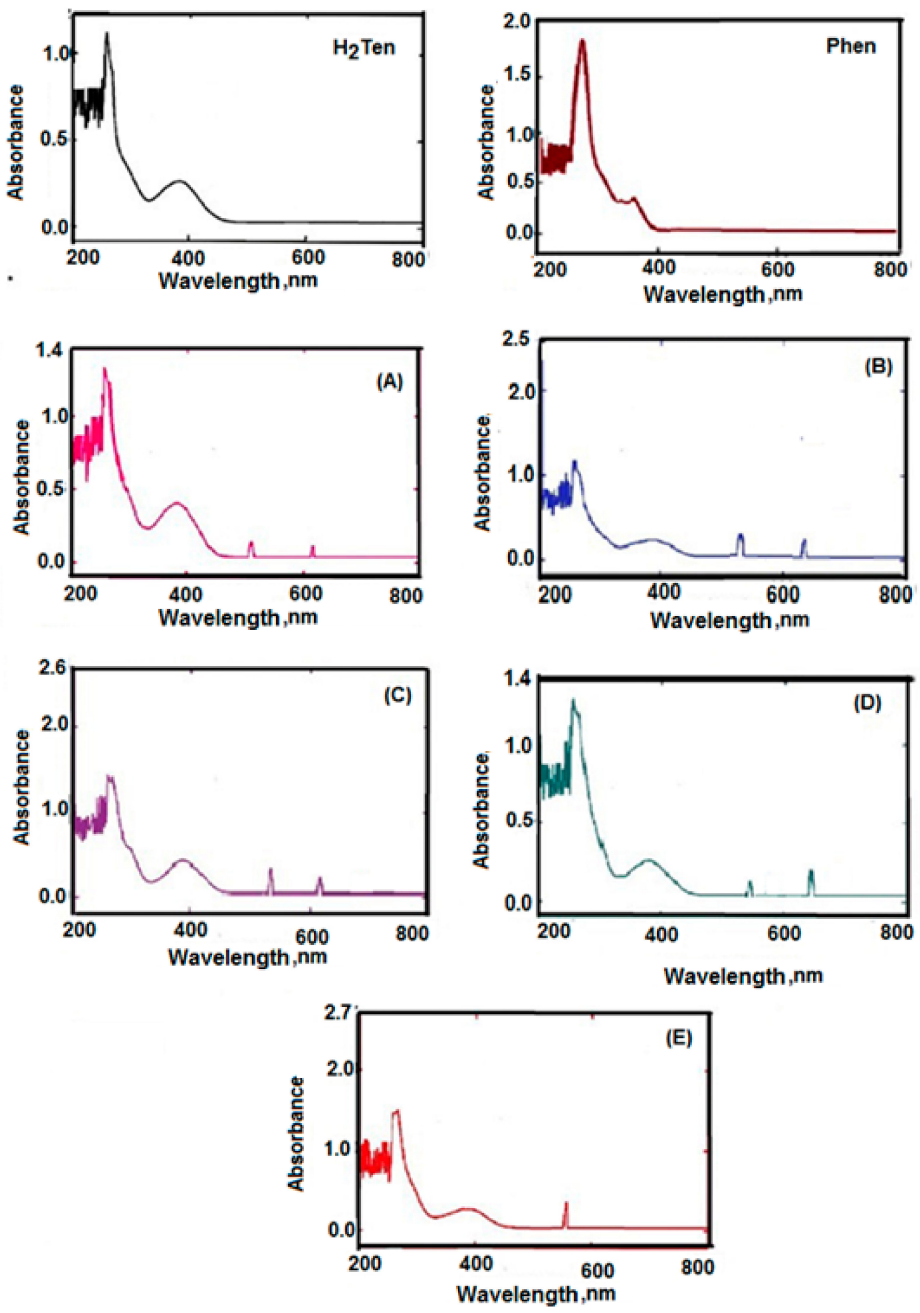
Electronic absorption spectrum for H_2_Ten, Phen and their metal complexes. Where: H_2_Ten: Tenoxicam; Phen: 1,10-Phenanthroline; (**A**) Manganese complex; (**B**) Cobalt complex; (**C**) Nickel complex; (**D**) Cupper complex; (**E**) Zink complex.

Figure 1 displayed the UV-Vis spectra of the organized mixed ligand complexes (A), (B), (C), (D), and (E) in DMSO solvent in the region of 200–800 nm. Free H_2_Ten ligand showed off three maximum absorption bands at 241, 257, and 383 nm which may also be assigned to π- π* and n-π* transitions, respectively (Table 2) [30]. Phen accords two bands at 273 and 350 nm, which assigned to π-π* and n-π* transitions within C=N group, respectively [28,31,32]. When ligands were chelating with metal ions, there were motivating change in the electronic properties of the system. New bands can be detected in the visible region due to the charge transfer interaction of ligand to metal (L→M) or metal to ligand (M→L) in the 510–560 nm range, which can help us to get a lot of information about the geometric structure of mixed ligand complexes [32]. The absorbance spectrum of the complex (A) shows a band identified at 615 nm that can be assigned to the transition ^6^A_1g_→^4^T_1g_(4G) [32]. For (B) complex spectrum showed a band at 620 nm that can be assigned to the transition 4T_1g_(F) →4T_1g_ (P) in favor of octahedral geometry [33]. The (C) complex showed absorption band at 605 nm, which may be assigned to ^3^A_2g_→^3^T_1g_(P) transition and supporting distorted octahedral geometry [33]. The band observed at 630 nm for(D) complex may be assigned to ^2^B_1g_^2^E_g_ transition with the magnetic moment (1.70 B.M.) is very close to spin value (1.73 B.M.) expected for an octahedral geometry [34].

### 2.3. ^1^H NMR Spectra

**Figure 2:**
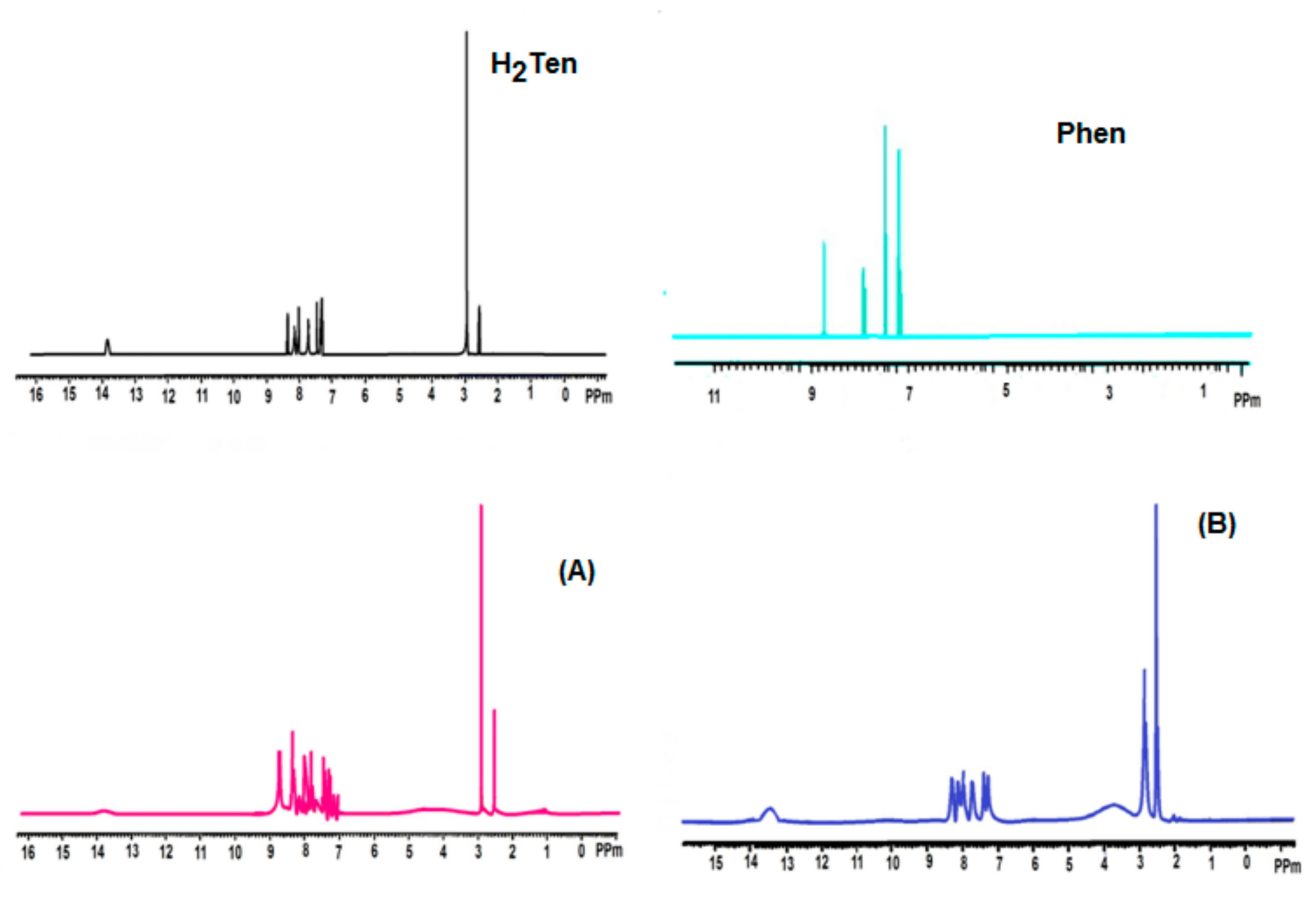

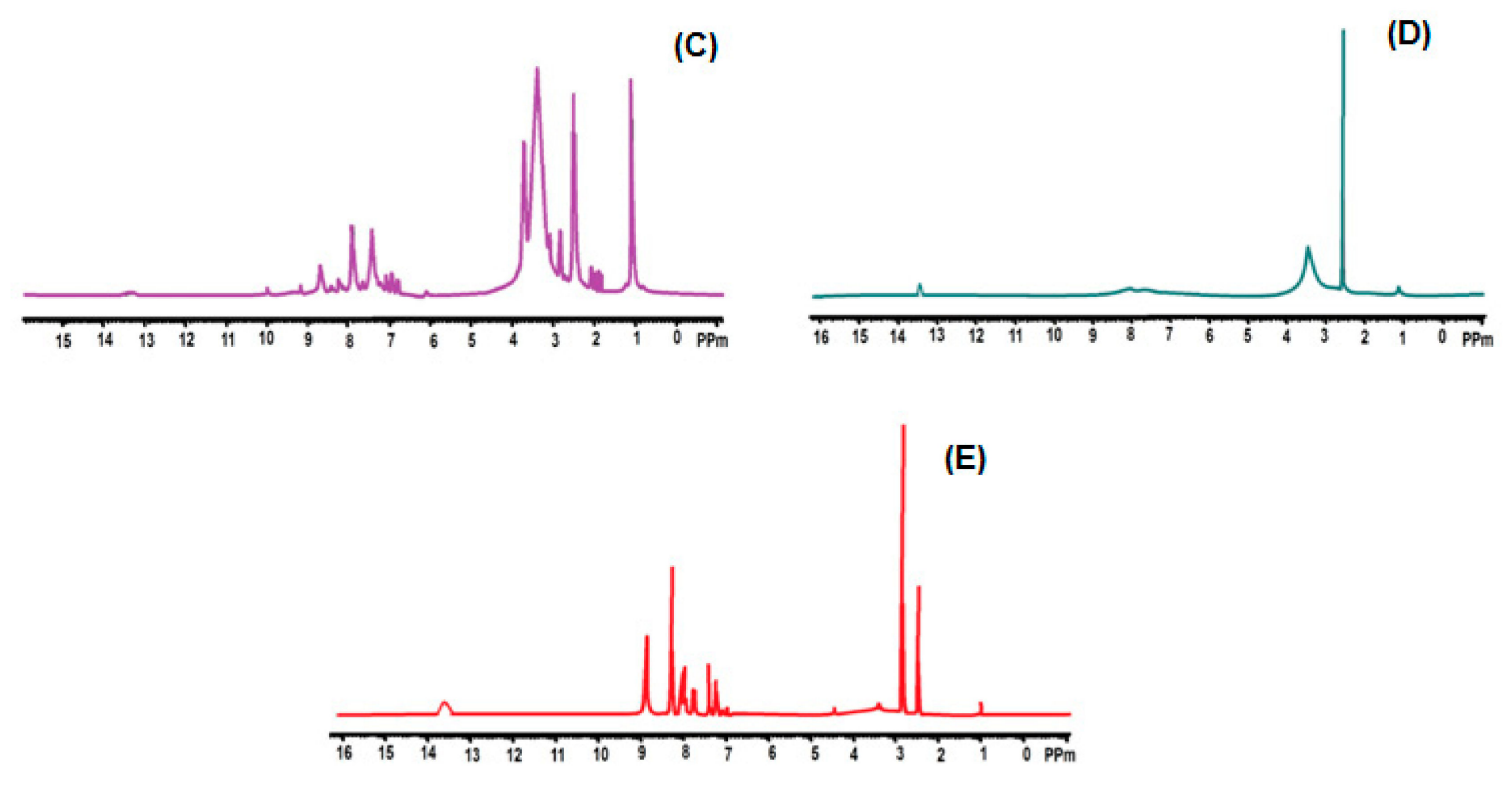
^1^H NMR spectrum for H_2_Ten, Phen and their metal complexes. Where: H_2_Ten: Tenoxicam; Phen: 1,10-Phenanthroline; (**A**) Manganese complex; (**B**) Cobalt complex; (**C**) Nickel complex; (**D**) Cupper complex; (**E**) Zink complex.

The assignments of the most important signals in the ^1^H NMR spectra of H_2_Ten, Phen, and their metal complexes are recorded in Table S2. H_2_Ten confirmed singlet peak at 13.80 ppm for OH enolate (Figure 2), aromatic-H signals at 7.29–8.32, at 8.34 for- NH amine, and singlet at 2.51 for –CH_3_ methyl group. The signals at 7.26–8.81 ppm (m, Ar-H) assigned to aromatic protons observed in Phen ligand. All signals in the complexes move to the lower fields increasingly compared to H_2_Ten and Phen ligands. Water molecules were assigned signs with points in the range of 3.09–4.46 ppm with an integration corresponding to protons in all metal complexes [35]. By matching the main peaks of H_2_Ten and Phen with the complexes, the difference in the chemical substance is related to the coordination of the H_2_Ten pyridine nitrogen that modifies the electronic environment around the neighborhood proteins (Figure 2). In addition, the change in aromatic protons can be the donation of electronic density of metal ions in chelating [36]. Similar ^1^H NMR spectral studies of paramagnetic complexes have been advanced in their reports on paramagnetic Cu(II) complexes. In addition, the ^1^H NMR spectra of paramagnetic Mn(II), Co(II), Ni(II), and Cu(II) complexes have been studied [37,38].

### 2.4. Mass Spectra

**Scheme 3:**
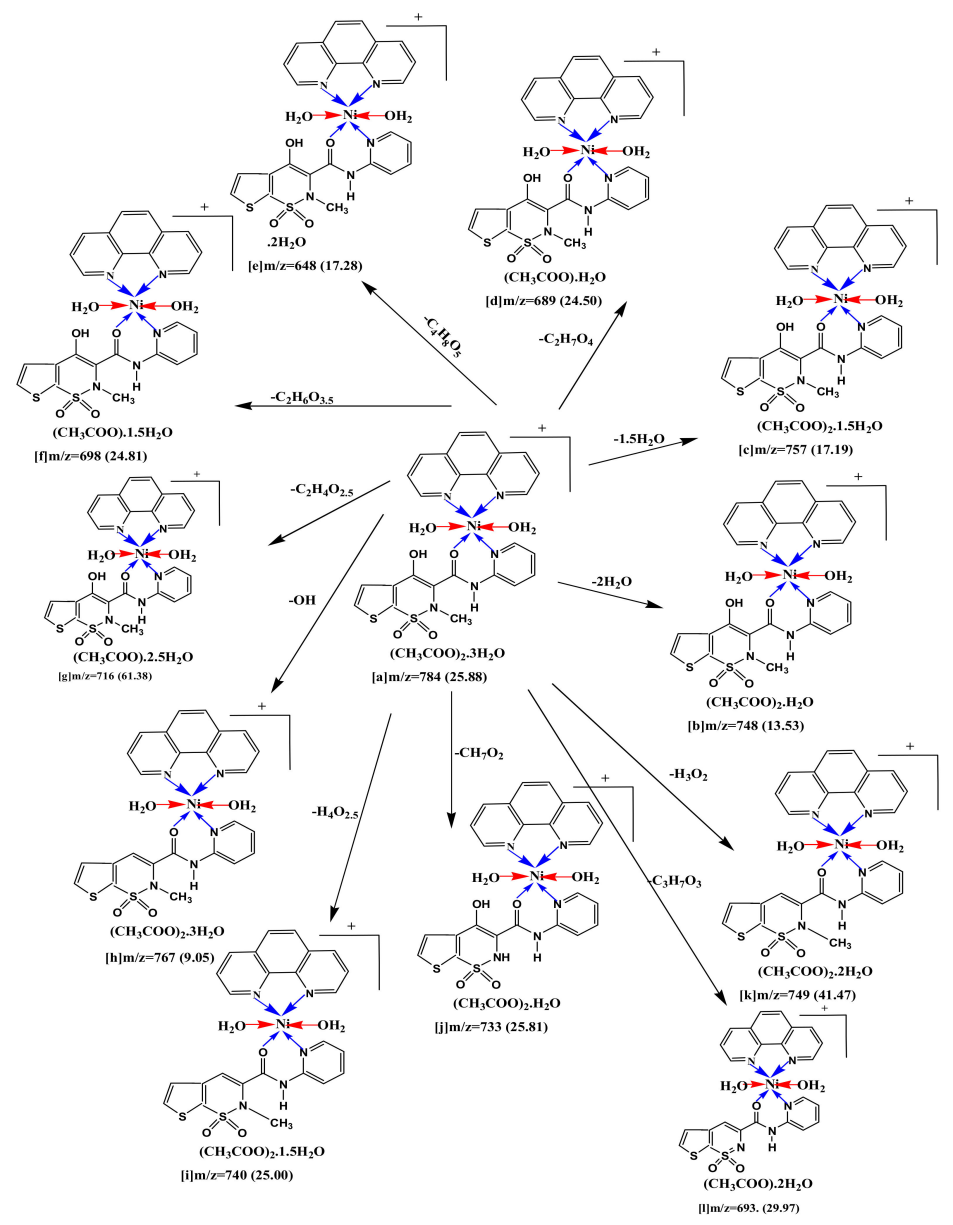
Fragmentation pattern of (C) complex.

The proposition molecular formulas of metal complexes were confirmed by paralleling their molecular formulas weights with *m/z* values. The mass spectra of complexes (Figure S2) displayed that the molecular ion peaks at *m/z* = amu with the calculated molecular weights of 714 (42.54%), 700 (9.06%), 748 (25.88%), 723 (55.05%), and 688 (17.59) for (A), (B), (C), (D), (E), and (F) complexes, respectively. This information is in precise settlement with the respective molecular formulation of metallic complexes. The fragmentation sample and mass spectrum of (C) complicated as representative example, is depicted in Figure S2. The molecular ion peak [a] appeared at *m/z* = 784 (25.88%) loses 2H_2_O to give [b] at *m/z* = 748 (13.53%) and it loses 1.5H_2_O to give [c] at *m/z* = 757 (17.19%). The molecular ion peak [a] loses C_2_H_7_O_4_ to give [d] at *m/z* = 689 (24.50%) and it loses C_4_H_8_O_5_ to give [e] at *m/z* = 648 (17.28%). The molecular ion peak [a] loses C_2_H_6_O_3.5_ to give fragment [f] at *m/z* = 698 (24.81%) and it loses C_2_H_4_O_2.5_ to give fragment [g] at *m/z* = 716 (61.38%). The molecular ion peak [a] loses OH to give [h] at *m/z* = 767 (9.05%) and it loses H_4_O_2.5_ to give [i] at *m/z* = 740 (25.00%), [a] loses CH_7_O_2_ to give [j] at *m/z* = 733 (25.81%) and it loses H_3_O_2_ to give [k] at *m/z* = 749 (41.47%). In addition, [a] loses C_3_H_7_O_3_ to give [l] at *m/z* = 693 (29.97%) (Scheme 3).

### 2.5. Thermal Analysis Studies

**Figure 3:**
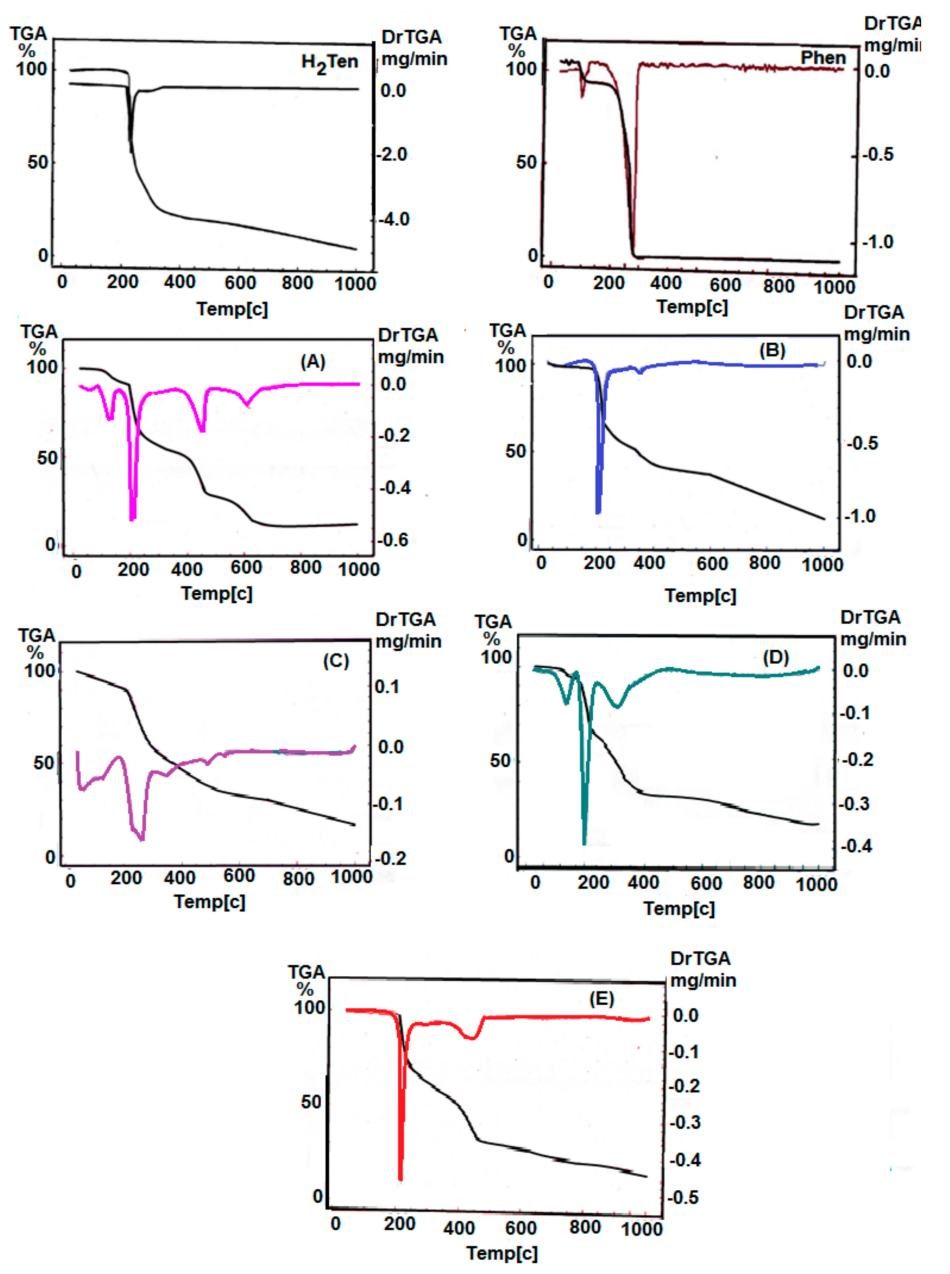
TGA and DTG diagram for H_2_Ten, Phen and their metal complexes. Where: H_2_Ten: Tenoxicam; Phen: 1,10-Phenanthroline; (**A**) Manganese complex; (**B**) Cobalt complex; (**C**) Nickel complex; (**D**) Cupper complex; (**E**) Zink complex.

To assert the structures and compositions thermogravimetric (TGA) and differential thermogravimetric (DTG) analysis were executed for mixed ligand complexes (A), (B), (C), (D), and (E). The measurements were executed below nitrogen surroundings in the temperature range of 25–1000 °C. Their consultant thermo grams are refined in Figure 3. Possible thermal decay patterns for complexes are revealed in Table 3. The study of the thermo grams of the complexes provision several observations. The establish weight losses have been in settlement with the theoretical losses. The thermal evaluation of H_2_Ten has been accounted in the literature [28,39]. TG curve exhibits Phen decomposed at two steps with mass loss 9.02 and 90.98% corresponding to H_2_O and 4C_2_H_2_+C_4_H_2_+N_2_, respectively (Table 3). TGA of (A), (B), (C) and (D) complexes exhibited three decomposition steps. The first step occur at 124, 63, 120 and 129 ^ο^C as maximum temperatures, respectively, and the mass loss lattice water were 5.04, 2.50, 6.85 and 4.91% respectively. The second step for the four complexes occurred at 208, 212, 258 and 203 ^ο^C as maximum temperatures, respectively, with the weight loss 30.30, 30.80, 27.35 and 29.80% corresponding to the loss of 6C_2_H_2_+2NO molecules for the four complexes. The third step with 447, 611, 251, 347, 349 and 309 ^ο^C maximum temperatures and a weight loss 53.15, 54.76, 52.92 and 47.48% corresponding to the loss of H_2_Ten molecule giving MnO+C, CoO+C, NiO+2C, and CuO+4C as final products. TGA curve of the (E) complex exhibit two main decomposition steps. The first step occurs at 217 ^ο^C maximum temperature with a weight loss of 31.32%, corresponding to the loss of Phen and the coordinated water molecules. The second step with 439 ^ο^C maximum temperature and a weight loss of 53.98%, corresponding to the loss of H_2_Ten molecule giving ZnO+2C as final product.

Based on thermal curves, two techniques [40,41] were used to assess kinetic thermodynamics parameters activation energy (E*), the enthalpy of activation (ΔH*), the entropy of activation (ΔS*), Gibbs, free energy change of the decomposition (ΔG*). The parameters (E*, A, ΔH*, ΔS*, and ΔG*) associated to the complexes have been evaluated graphically (Figure S3), and the evaluated data are tabulated in Table 4. The ΔG* values were positive for the complexes, enlightening that the thermal decomposition was a non-spontaneous process, the complexes the complexes being thermally stable [42]. The ΔS* values were instituted to be negative. The negative values of the ΔS* fixed that the activated complexes had a more ordered structure than the reactants, or that the reaction used to be slower than the normal [42]. The positive values of ΔH* intended that the decomposition manner was once endothermic.

### 2.6. Antimicrobial Efficiency

#### 2.6.1. Antifungal Activity

The possible fungicidal activity of tenoxicam and its metal complexes was determined against three serious phytopathogens (*P. digitatum*, *C. acutatum* and *M. fructicola*) compared to positive control Azoxystrobin (0.8 µL/mL) as demonstrated in Table 5. Generally, all tested substances showed antifungal effect in a dose dependent manner.

In particular, the highest significant antifungal effect against *P. digitatum* has been observed in case of H_2_Ten (100 ppm), (A: 100 and 50 ppm), (B: 100 ppm), (C: 100 ppm), and (E: 100 ppm), whereas the lowest significant effect has been observed in case of (C: 50 ppm), compared to positive control. Regarding *C. acutatum*, the highest significant effect has been observed in case of (A: 100 and 50 ppm) and (C: 100 ppm) insignificantly with positive control, whereas the lowest significant effect has been observed in case of H_2_Ten (50 ppm). On the other hand, the highest significant antifungal effect against *M. fructicola* has been observed in case of (A: 100 and 50 ppm), (B: 100 ppm), and (C: 100 ppm), whereas the lowest significant effect has been observed in case of H_2_Ten (50 ppm) and (E: 50 ppm).

#### 2.6.2. Antibacterial Activity

**Figure 4:**
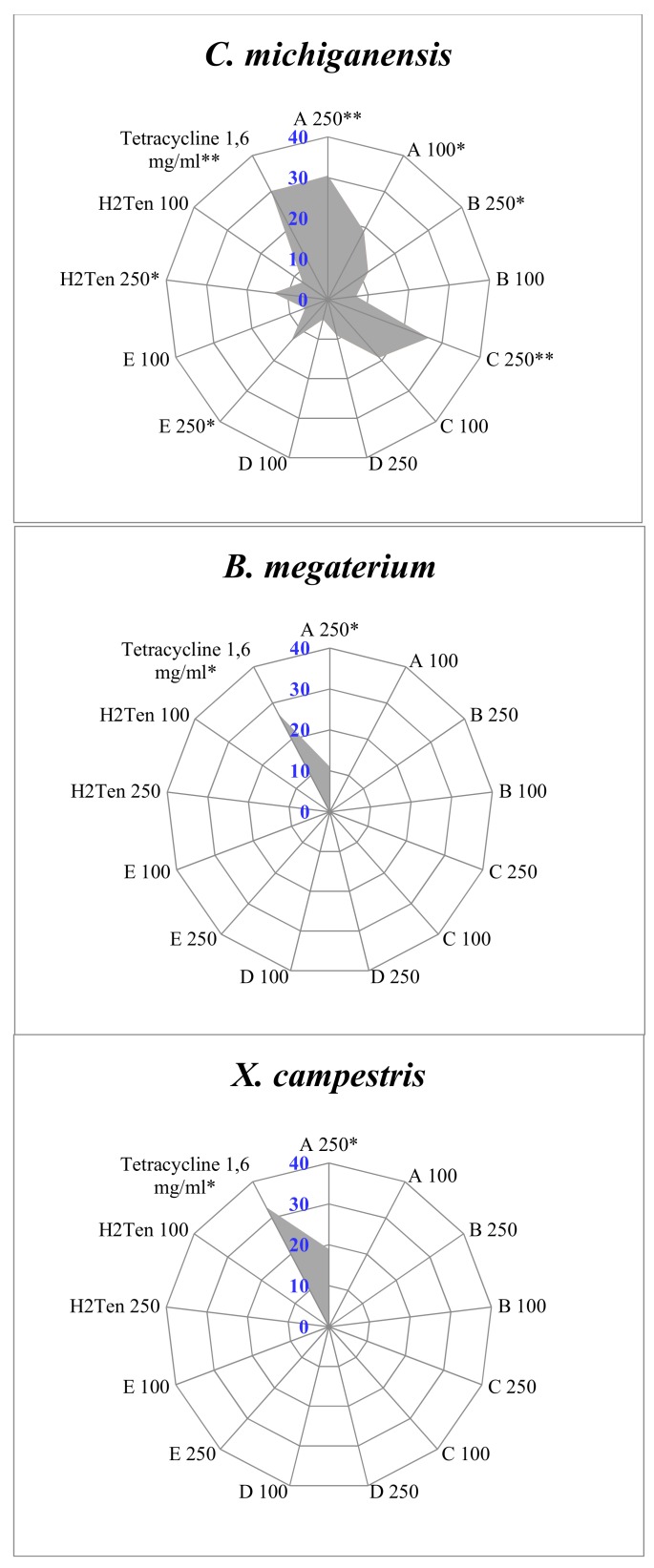
Antimicrobial activity of H_2_Ten and its metal complexes. Where: H_2_Ten: Tenoxicam; A: Manganese complex; B: Cobalt complex; C: Nickel complex; D: Cupper complex; E: Zink complex; Tetracycline: 1.6 mg/mL. Values are recorded as the mean of diameter of inhibition zones (mm) from three replicates ± SDs. (*): statistically significant at 𝑃 < 0.05: (**): statistically significant at 𝑃 < 0.01.

The results of the antibacterial test showed that the tested ligand and its metal complexes were able to inhibit the growth of *X. campestris* in a dose dependent manner. In particular, the highest significant effect was observed in the case of (A and C) at 250 ppm insignificantly with Tetracycline (1600 μg.mL^−1^), whereas the lowest effect was observed in the case of (E) at 100 ppm (Figure 4). Regarding *C. michiganensis* and *B. megaterium*, Tetracycline (1600 μg.mL^−1^) showed the highest significant effect, followed by (A) at 250 ppm, whereas there is no effect observed for other studied compounds.

#### 2.6.3. Mode of Action

The antimicrobial activity of the studied metal complexes could be due to the chemical structures of free ligand itself and the toxic nature of some metal ions [43,44]. On the other hand, the attachment between the parent ligand and the metal ions through the chelation process has reduced the polarity of some metal ions by sharing its positive charge with the donor groups, and possibly the π-electron delocalization within the whole chelate ring system [44]. Based on the above discussed mechanism, it is strongly hypothesized that the chelation process was able to increase the permeability of the microbial cell walls and the lipophilic nature of metal complexes, which lead to their penetration to the peptidoglycan layer of the plasma membrane, and later on lead to the complete cell death [43,45].

### 2.7. Phytotoxicity Assay

The studied compounds exhibited highly phytotoxic effect against all tested plants (*S. lycopersicum* and *L. sativum*) at all tested concentrations. In particular, the tested free ligand and their metal complexes negatively affected the seed germination (SG) and radical elongation (RE) of the above-mentioned tested plants compared to the control treatment. On the other hand, H_2_Ten at 100 ppm showed moderate phytotoxic effect similar to the control treatment. The obtained results indicated the need for future research on the possible use of the newly prepared metal complexes in controlling different phytopathogens, and also in the pharmaceutical industry.

### 2.8. Cytotoxicity Screening

**Figure 5:**
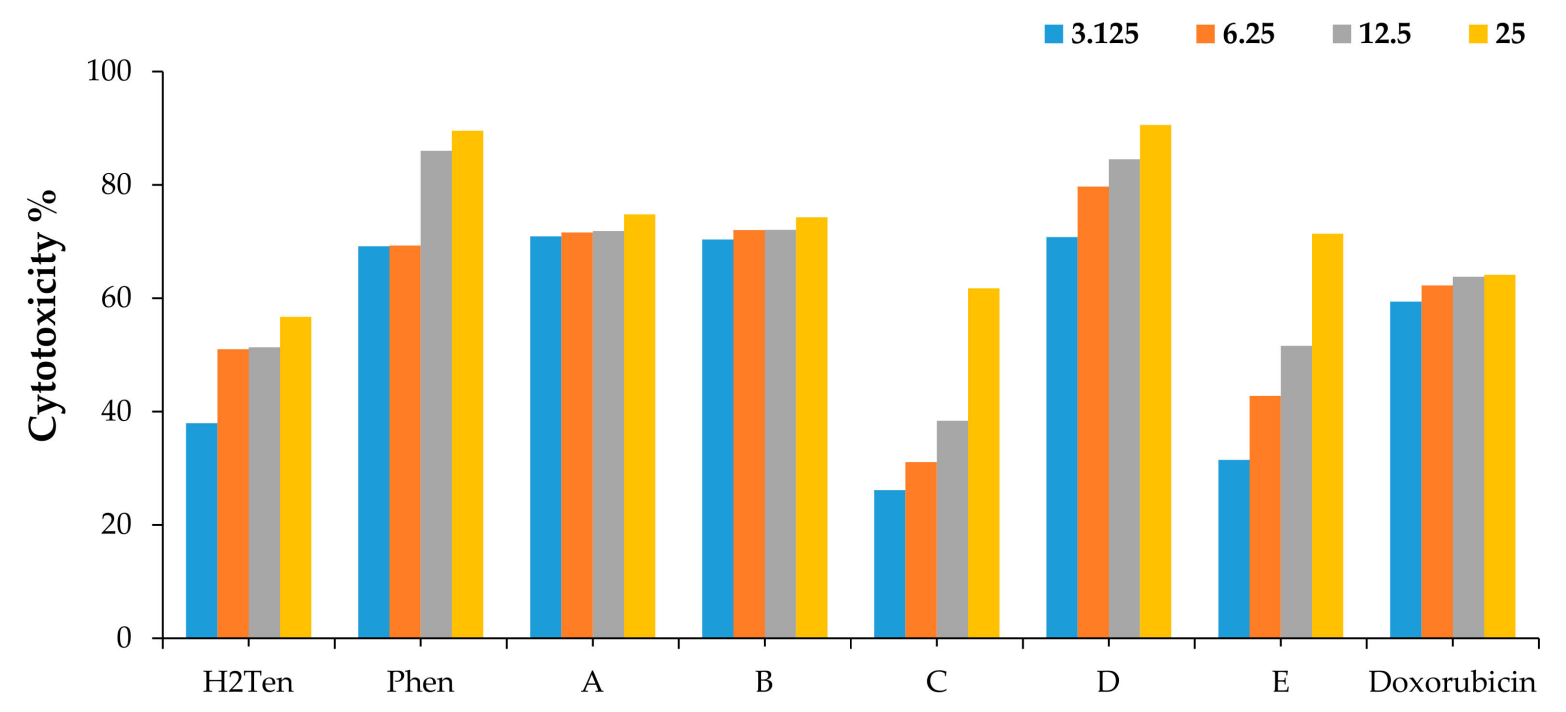
Dose dependent cytotoxic activities of H_2_Ten, Phen and their metal complexes against HCT-116 cancer cells according to the MTT assay. Where: 3.12, 6.25, 12.5, and 25.0 µM are the concentrations, diluted in DMSO, used for the MTT assay.

**Figure 6:**
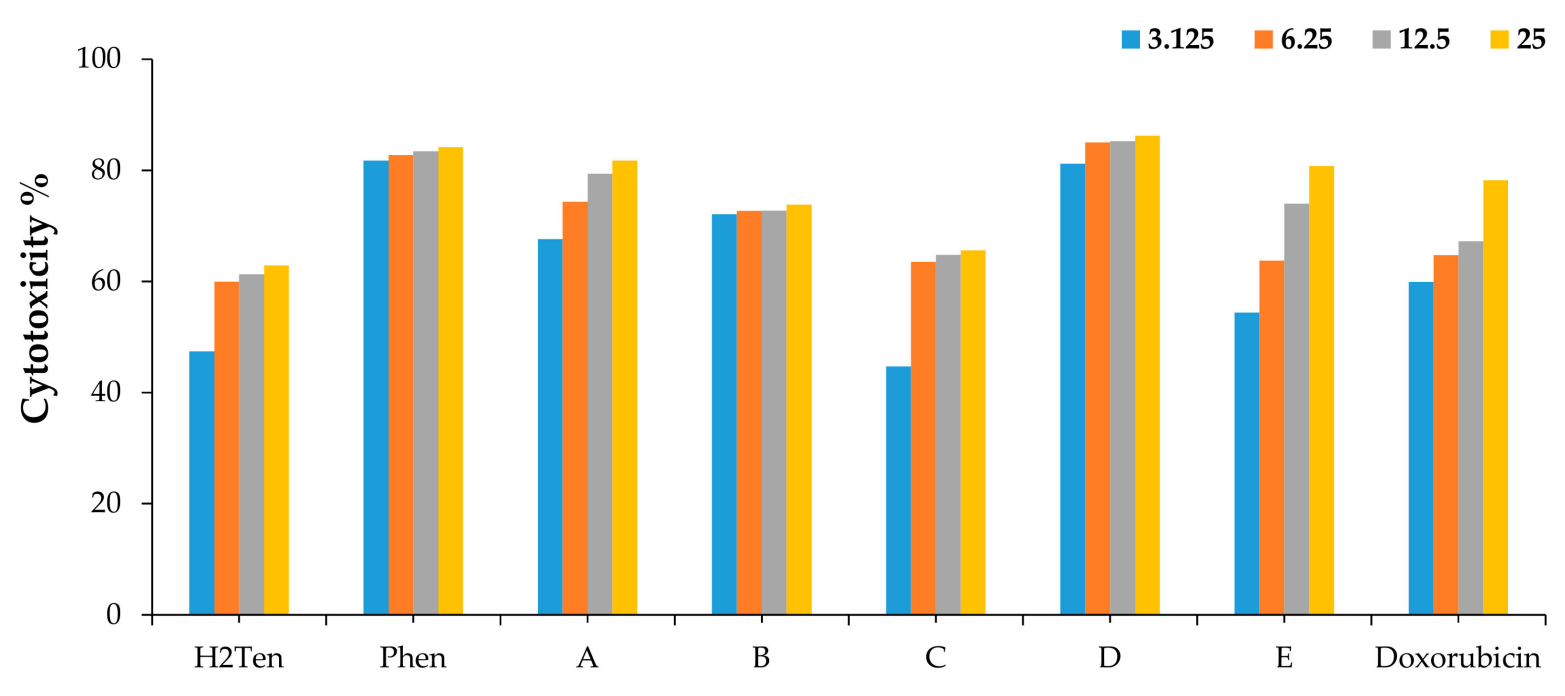
Dose dependent cytotoxic activities of H_2_Ten, Phen and their metal complexes against MCF-7 cancer cells according to the MTT assay. Where: 3.12, 6.25, 12.5, and 25.0 µM are the concentrations, diluted in DMSO, used for the MTT assay.

**Figure 7:**
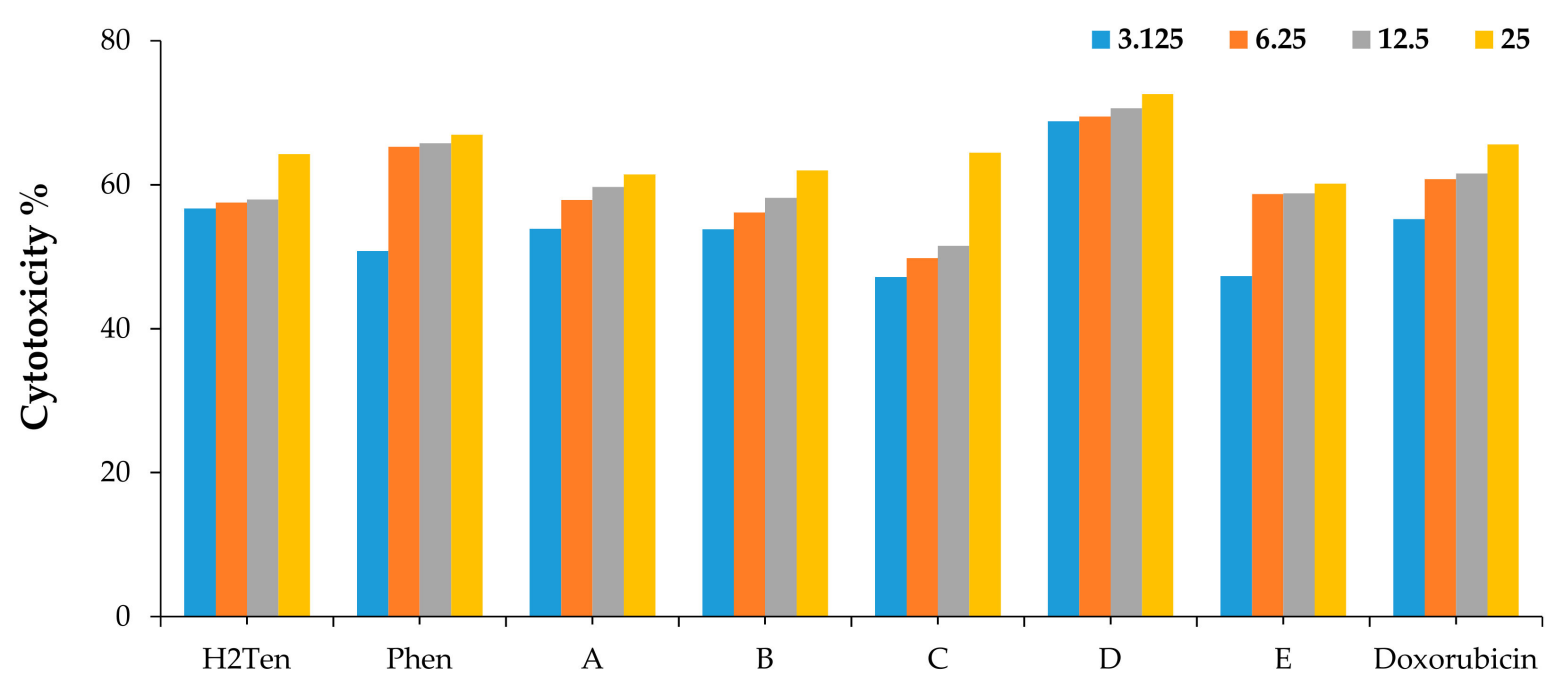
Dose dependent cytotoxic activities of H_2_Ten, Phen and their metal complexes against HepG2 cancer cells according to the MTT assay. Where: 3.12, 6.25, 12.5, and 25.0 µM are the concentrations, diluted in DMSO, used for the MTT assay.

The compounds were examined in vitro for their activity against HCT-116, HepG2, and MCF-7 human cancer cells using the MTT assay. The percentages of intact cells were calculated and compared to those of the control. Activities of these compounds against the three carcinoma cell lines were compared to the activity of doxorubicin as well (Table 6). All compounds suppressed the three cancer cells in a dose-dependent manner (Figure 5, Figure 6 and Figure 7). In case of HCT-116 human colorectal carcinoma cells, the data showed that compounds Phen, (A), (B), and (D) more potent cytotoxic compounds, and the compounds H_2_Ten, (C), and (E) are slightly less active compared to doxorubicin. For MCF-7 human breast cancer cells, the compounds Phen and (A), (B), (E), and (D) are more potent cytotoxic compounds, while the two compounds H_2_Ten and (C) are slightly less active compared to doxorubicin. In case of HepG2 human liver carcinoma cells, two compounds Phen and (D), were more potent than the reference drug (H_2_Ten) and the rest of the compounds (A), (B), (C), and (E) were slightly less active against HepG2 compared to doxorubicin.

## 3. Experimental

### 3.1. Chemicals, Materials, and Biological Species

The analytical reagents grade tenoxicam has been purchased from Egyptian International Pharmaceutical Industries Company (EIPICO). The chemicals 1,10-phenanthroline monohydrate, ethanol, MnCl_2_.H_2_O, CoCl_2_.6H_2_O, Ni(CH_3_COO)_2_.2H_2_O, CuCl_2_.2H_2_O, and ZnCl_2_.H_2_O (from Fluka and Aldrich Chemical Company) were used without further purification. HCT-116 (human colorectal carcinoma), HepG2 (human hepatocellular carcinoma) and MCF-7 (human breast adenocarcinoma) cell lines were purchased from the American Type Culture Collection (Rockville, MD, USA).

### 3.2. Synthesis of New Metal Complexes

The Pale orange [Mn(H_2_Ten)(Phen)(H_2_O)_2_]Cl_2_.2H_2_O, complex (A) was prepared by mixing 1 mmol (0.337 g) of H_2_Ten and 1 mmol (0.156 g) of Phen in 30 mL absolute ethanol with 1 mmol (0.14393 g) of MnCl_2_.H_2_O in 20mL ethanol. The mixture was refluxed for 3 h and the precipitate was filtered off and dried under vacuum over anhydrous CaCl_2_. The green, Pale green, Dark green and Pale yellow solid complexes [Co(H_2_Ten)(Phen)(H_2_O)_2_]Cl_2_.H_2_O, (B), [Ni(H_2_Ten)(Phen)(H_2_O)_2_](CH_3_COO)_2_.3H_2_O, (C), [Cu(H_2_Ten)(Phen)(H_2_O)_2_]Cl_2_.2H_2_O, (D), and [Zn(H_2_Ten)(Phen)(H_2_O)_2_]Cl_2_, (E) were prepared in a similar method described above by using CoCl_2_.6H_2_O, Ni(CH_3_COO)_2_.2H_2_O, CuCl_2_.2H_2_O, and ZnCl_2_.H_2_O, respectively.

### 3.3. Instruments

FT-IR spectra in KBr discs were recorded in the range from 4000–400 cm^−1^ with FT-IR 460 PLUS Spectrophotometer. Electronic spectra were carried out using UV-3101PC Shimadzu. The absorption spectra were recorded as solutions in DMSO-d_6_. ^1^H NMR spectra were recorded on Varian Mercury VX-300 NMR Spectrometer using DMSO-d_6_ as solvent. The elemental analyses were performed using a Perkin Elmer 2400 CHN elemental analyzer. TGA and differential thermal gravimetric analysis (DTG) measurements were done under N_2_ atmosphere within the temperature range from room temperature to 1000 ^ο^C using TGA-50H Shimadzu, the mass of sample was accurately weighted out in an aluminum crucible. The percentage of the metal ions were determined gravimetrically by transforming the solid products into metal oxide, and also determined by using atomic absorption method. Spectrometer model PYE-UNICAM SP 1900 fitted with the corresponding lamp was used for this purposed Mass spectra were recorded on GCMS-QP-1000EX Shimadzu (ESI-70ev) in the range from 0–1090. Room temperature magnetic susceptibilities of the powdered samples were done on a Sherwood scientific magnetic balance using Gouy balance at room temperature using Hg[Co(CSN)_4_] as calibrant. Melting points were recorded on a Buchi apparatus. All measurements were carried out at ambient temperature with freshly prepared solutions. The molar conductance of 1 × 10^−3^ M solutions of the ligands and their complexes in DMF was measured at room temperature using CONSORT K410.

### 3.4. Antimicrobial Investigation

#### 3.4.1. Antifungal Activity

Tested fungal isolates. The tested phytopathogenic fungi were monoconidic isolates of *M. fructicola, P. digitatum* and *C. acutatum.* They were stored at 4 °C as pure cultures in the mycotheca of the School of Agricultural, Forestry, Food, and Environmental Sciences (SAFE) of Basilicata University, Potenza, Italy. The fungal species were recultured on potato dextrose agar (PDA) at 24 ± 2 °C. All tested fungi were previously identified by morphological and molecular methods based on polymerase chain reaction (PCR) and sequences analysis using Basic Local Alignment Search Tool software (BLAST- Bethesda, Rockville, MD, USA).

Fungicidal assay. The possible fungicidal activity of the studied ligands and their metal complexes was evaluated following incorporation method at two different concentrations (250 and 250 and 125 µg/mL) into potato dextrose agar (PDA) medium at 45 °C [46]. Fungal disk (0.5 cm) from 96 h fresh culture was inoculated in the center of each Petri dish. All plates were incubated at 22 ± 2 °C for 96 h in darkness conditions and the diameter of the fungal mycelium was measured in mm. The PDA plates without any treatment were inoculated with fungal disks as control. Fungi toxicity was expressed as percentage of growth inhibition (PGI) and calculated according to the Equation (1) [47] compared to Azoxystrobin (0.8 µL/mL), a large spectrum fungicide, as control according to the international limit of microbicide standards:

(1) $$\mathrm{PGI}\;\left(\%\right)=\;\frac{100\;\times \;\left(\mathrm{GC}-\mathrm{GT}\right)}{\mathrm{GC}}$$

where PGI is the percentage of growth inhibition, GC is the average diameter of fungal mycelium in PDA (control), and GT is the average diameter of fungal mycelium on the EO-treated PDA dish.

#### 3.4.2. Antibacterial Activity 

Tested bacterial isolates. The tested bacterial strains were *C. michiganensis*, *X. campestris* and *B. megaterium,* have been conserved as pure cultures in the collection of SAFE, University of Basilicata, Potenza, Italy.

Bactericidal assay. The antibacterial activity test has been carried out following the disc diffusion method [48]. The bacterial suspension of each strain was prepared in sterile distilled water incorporated into soft agar (0.7%) adjusted by Turbidimetry (Biolog, USA) at 10^8^ colony form unit (CFU) mL^−1^ corresponding to 0.2 nm optical density (OD). Four mL of soft agar and bacterial suspension (9:1 *v/v*) was poured into Petri dish (Ø 90 mm) containing 10 mL KB media. Blank Discs (Ø6 mm) (OXOID, Milan, Italy) were placed over KB-plate surfaces and 20 µL from each compound were applied over the discs at the following concentrations: 125 and 250 µg/mL. Tetracycline was used as positive control at 1600 μg mL^−1^. All plates were incubated at 37 ± 2 °C for 24 hrs and the bactericidal activity has been evaluated by measuring the diameter of inhibition zones (mm). All tested treatments have been carried out in triplicates.

### 3.5. Phytotoxicity Assay

A bioassay based on SG and RE was carried out to evaluate the possible phytotoxic effect of the studied compounds on *S. lycopersicum* and *L. sativum* seeds [43,49]. Seeds were sterilized in 3% H_2_O_2_ solution for 1 min and then were rinsed twice with deionized sterile water (dH_2_O). Seeds were placed either in dH_2_O (control) or the above mentioned compounds and were shaken gently for 2 hrs. The tested concentrations were 100, 250, 500, 1000, and 2000 ppm. All seeds were subsequently transferred into 15 mm × 100 mm Petri dishes containing one piece of filter paper (Ø90 mm, Whatman No.1). Ten seeds of each species were evenly spaced on top of the filter paper and moistened with 2 mL of dH_2_O, or different compounds, and sealed with Parafilm. All petri dishes were incubated in a growth chamber at 24 ± 2 °C with 60% relative humidity in dark conditions for 96 h. The number of germinated seeds was counted and the radical length was measured in cm. The experiment has been conducted in triplicate and the germination index (G.I.) was calculated using the Equation (2):

G.I. % = [(SG_t_ × RE_t_)/(SG_c_ × RE_c_)] × 100 (2)

where: GI: germination index; SG_t_: average number of germinated treated seeds; RE_t_: average radical elongation for treated seeds; SG_c_: average number of germinated seeds for dH_2_O control; RE_c_: average radical elongation for dH_2_O control. Data are expressed as the mean ± SDs for the number of germinated seeds, radical elongation and germination index. Data were analyzed using SPSS statistical program with Tukey test at *P* < 0.05.

### 3.6. Cytotoxic Activity

Cell culture of HCT-116 (human colorectal carcinoma), HepG2 (human hepatocellular carcinoma), and MCF-7 (human breast adenocarcinoma) cell lines were purchased from the American Type Culture Collection (Rockville, MD) and maintained in DMEM medium, which was supplemented with 10% heat-inactivated fetal bovine serum (FBS), 100 U/mL penicillin, and 100 U/mL streptomycin. The cells were grown at 37 °C in a humidified atmosphere of 5% CO_2_.

#### MTT Cytotoxicity Assay

The cytotoxicity activity against HCT-116, HepG2, and MCF-7 human cancer cell lines was estimated using the 3-[4,5-dimethyl-2-thiazolyl)-2,5-diphenyl-2H-tetrazolium bromide (MTT) assay, which is based on the cleavage of the tetrazolium salt by mitochondrial dehydrogenases in viable cells [50,51,52]. Cells were dispensed in a *96* well sterile microplate (5 × 10^4^ cells/well), and incubated at 37 °C with series of different concentrations (3.12, 6.25, 12.5 and 25.0 µM), in DMSO, of each tested compound or Doxorubicin (positive control) for 48 h in a serum free medium prior to the MTT assay. After incubation, media were carefully removed and 40 µL of MTT (2.5 mg/mL) were added to each well and then incubated for an additional 4 h. The purple formazan dye crystals were solubilized by the addition of 200 µL of DMSO. The absorbance was measured at 590 nm using a Spectra Max Paradigm 1` Multi-ode micro plate reader. The relative cell viability was expressed as the mean percentage of viable cells compared to the untreated control cells. All experiments were performed in triplicate on three different days. Results were represented as mean ± SD. The half maximal inhibitory concentration (IC_50_) were calculated by probit analysis by SPSS Inc. software (IBM Corp, Armonk, NY, USA).

## 4. Conclusions

The following metal ions Mn(II), Co(II), Ni(II), Cu(II), and Zn(II) reacted with H_2_Ten and Phen forming new complexes. The structures of the new synthesized complexes were investigated using various spectroscopic techniques. The obtained data indicated that H_2_Ten and Phen ligands worked as bidentate chelates and coordinated to the metal via i) the oxygen atom of the amide group; ii) the nitrogen atom of the pyridyl ring; and iii) two atoms of nitrogen of Phen. Results supported the hypothesized octahedral structure of the new metal complexes. Thermal analyses showed a significant mass decrease due to the loss of water molecules, either initially or during successive steps. On the other hand, Phen and (D) compounds were more potent than the parent ligand (H_2_Ten) against HepG2 human liver carcinoma cells, whereas the other compounds (A), (B), (C), and (E) were slightly less active compared to doxorubicin. Regarding the antimicrobial effects, the obtained results showed that the tested ligand and its metal complexes were able to inhibit the majority of tested microorganisms in a dose dependent manner, and this effect could be due to the chemical structures of free ligand itself and the toxic nature of some attached metal ions.

## Figures and Tables

**Table 1:** Selected infrared wave numbers (cm^−1^) for H_2_Ten, Phen and their metal complexes

Compounds	ν(O-H);enolate and H_2_O;	ν(C=O); amide	ν(C=N) in pyridyl H_2_Ten	ν(C=N) in pyridyl Bipy	ν_as_(so_2_)	ν_s_(so_2_)	ν(M-O) and *ν*(M-N)
H_2_Ten	3432s	1635vs	1599s		1327s	1040 m	-
Phen	3380 mbr	-	-	1586 ms	-	-	-
(A)	3402 m,br	1600 s	1549 m	1510 vw	1325 m	1050vw	608 m 526 w 424 vw
(B)	3436 s	1600 s	1551 m	1520 w	1328 s	1042 w	624 m 582 m 528 m
(C)	3387 s,br	1627 vs	1592 s	1524 m	1325 m	1052 vw	626 vw 586 m 543 vw
(D)	3423 m,br	1640 vs	1533 w	1501 sh	1326 vs	1037 vs	617 m 530 w 423 w
(E)	3429 m,br	1600 vs	1547 m	1510 w	1328 s	1041 vw	627 w 548 vw 526 w

**Table 2:** UV–Vis. spectra for H_2_Ten, Phen and their metal complexes

Assignments (nm)	H_2_Ten	Phen	Mixed Ligand Complex With				
			(A)	(B)	(C)	(D)	(E)
π-π^*^ transitions	241 257	273	225 257	257	256	235 257	244 265
n-π^*^ transitions	383	350	383	382	386	380	382
Ligand-metal charge transfer	-	-	520	510	560	530	550
d-d transition	-	-	615	620	605	630	---

**Table 3:** The maximum temperature T_max_ (^ο^C) and weight loss values of the decomposition stages for H_2_Ten, Phen and their metal complexes

Compounds	Decomposition	T_max_ (^ο^C)	Weight Loss (%)		Lost Species
			Calc.	Found	
H_2_Ten (C_13_H_11_N_3_O_4_S_2_)	First step	228	92.89	93.83	5C_2_H_2_+2SO+CO_2_+0.5H_2_+1.5N_2_
	Second step		92.89	93.83	
	Residue		7.11	6.17	2C
Phen (C_12_H_10_N_2_O)	First step	95	9.07	9.02	
	Second step	278	90.93	90.98	H_2_O
	Total loss		100	100	4C_2_H_2_+C_4_H_2_+N_2_
	Residue				
(A) (MnC_25_H_27_N_5_O_8_S_2_Cl_2_)	First step	124	5.03	5.04	2H_2_O
	Second step	208	30.19	30.30	6C_2_H_2_+2NO
	Third step	447,611	53.19	53.15	5C_2_H_2_+NCCl+HCl+CO+N_2_+2SO
	Total loss		88.41	83.49	
	Residue		11.59	11.51	MnO+C
(B) (CoC_25_H_25_N_5_O_7_S_2_Cl_2_)	First step	63	2.56	2.50	H_2_O
	Second step	212	30.83	30.80	6C_2_H_2_+2NO
	Third step	251,347	54.22	54.76	5C_2_H_2_+2SO+NCCl+HCl+N_2_+CO
	Total loss		87.61	88.06	
	Residue		12.39	11.94	CoO+C
(C) (NiC_29_H_35_N_5_O_13_S_2_)	First step	63,120	6.89	6.85	3H_2_O
	Second step	258	27.54	27.35	6C_2_H_2_+2NO
	Third step	349	52.99	52.92	7C_2_H_2_+2SO+CO+NH_3_+2NO_2_
	Total loss		87.42	87.12	
	Residue		12.58	12.88	NiO+2C
(D) (CuC_25_H_27_N_5_O_8_S_2_Cl_2_)	First step	129	4.97	4.91	2H_2_O
	Second step	203	29.86	29.80	6C_2_H_2_+2NO
	Third step	307	47.55	47.48	4C_2_H_2_+2SO+NCCl+0.5Cl_2_+NO+NH_3_
	Total loss		82.39	85.30	
	Residue		17.61	17.81	CuO+4C
(E) (ZnC_25_H_23_N_5_O_6_S_2_Cl_2_)	First step	217	31.34	31.32	6C_2_H_2_+2NO
	Second step	439	53.39	53.98	5C_2_H_2_+2SO+Cl_2_+1.5N_2_+CO+0.5H_2_
	Total loss		84.73	85.30	
	Residue		15.27	14.70	ZnO+2C

**Table 4:** Thermal behavior and Kinetic parameters determined using Coats–Redfern (CR) and Horowitz–Metzger (HM) operated for H_2_Ten, Phen and their complexes.

Compounds	Decomposition Range (K)	T_s_(K)	Method	Parameter					R ^a^	SD ^b^
				E^*^ (KJ/mol)	A (s^−1^)	ΔS^*^ (KJ/mol.K)	ΔH^*^ (KJ/mol)	ΔG* (KJ/mol)		
H_2_Ten (C_13_H_11_N_3_O_4_S_2_)	401–525	501	CR HM	94.49 99.97	7.06 × 10^7^ 2.11 × 10^8^	−0.0989 −0.0898	90.32 95.81	139.90 140.8	0.989 0.984	0.154 0.183
Phen (C_12_H_10_N_2_O)	394–572	551	CR HM	117.83 146.78	2.03 × 10^9^ 7.97 × 10^11^	−0.0718 −0.0222	113.25 142.20	153.44 152.84	0.985 0.984	0.206 0.219
(A) (MnC_25_H_27_N_5_O_8_S_2_Cl_2_)	419–629	582	CR HM	85.59 77.30	3.5 × 10^6^ 1.65 × 10^6^	−0.1236 −0.1298	81.59 73.30	141.05 135.74	0.990 0.986	0.139 0.165
(B) (CoC_25_H_25_N_5_O_7_S_2_Cl_2_)	439–516	482	CR HM	128.73 124.95	4.37 × 10^11^ 3.73 × 10^11^	−0.0260 −0.027	124.72 120.94	137.28 134.13	0.998 0.997	0.052 0.067
(C) (NiC_29_H_35_N_5_O_13_S_2_)	440–741	531	CR HM	94.09 103.30	5.04 × 10^6^ 1.06 × 10^8^	−0.1213 −0.0960	89.68 98.89	154.13 149.88	0.991 0.985	0.132 0.176
(D) (CuC_25_H_27_N_5_O_8_S_2_Cl_2_)	419–507	476	CR HM	92.53 87.42	5.42 × 10^7^ 3.02 × 10^7^	−0.1007 −0.1055	88.58 83.46	136.52 162.23	0.992 0.989	0.124 0.148
(E) (ZnC_25_H_23_N_5_O_6_S_2_Cl_2_)	388–541	491	CR HM	96.50 86.20	3.92 × 10^7^ 1.05 × 10^7^	−0.1036 −0.1145	92.41 82.12	143.32 138.37	0.982 0.980	0.201 0.209

a: correlation coefficients of the Arrhenius plots. b: standard deviation.

**Table 5:** Antifungal activity of H_2_Ten and its metal complexes

Tested Substances		Fungal Growth Inhibition (%)		
	PPM	*P. digitatum*	*C. acutatum*	*M. fructicola*
H_2_Ten	250	87.5 ± 6.2a	32.5 ± 2.9c	67.5 ± 3.1b
	125	35.0 ± 3.1c	14.0 ± 2.1c	45.0 ± 2.5b
A	250	100.0 ± 0.0a	100.0 ± 0.0a	100.0 ± 0.0a
	125	100.0 ± 0.0a	100.0 ± 0.0a	100.0 ± 0.0a
B	250	87.5 ± 5.2a	54.0 ± 2.1b	81.0 ± 5.7a
	125	72.5 ± 4.3b	22.5 ± 1.2c	62.5 ± 5.4b
C	250	87.5 ± 5.6a	81.0 ± 5.8ab	77.5 ± 7.2b
	125	15.0 ± 2.1c	32.5 ± 3.1c	57.5 ± 2.1b
D	250	75.0 ± 3.1b	65.0 ± 1.4b	87.0 ± 3.5a
	125	36.5 ± 3.5c	27.0 ± 1.6c	61.0 ± 2.1b
E	250	87.5 ± 2.5a	62.5 ± 2.8b	65.0 ± 2.2b
	125	62.5 ± 6.4b	45.0 ± 3.4c	45.0 ± 5.6b
PDA	−ve cont.	0.0 ± 0.0e	0.0 ± 0.0d	0.0 ± 0.0c
Azoxy.	+ve cont.	58.1 ± 1.2b	92.5 ± 1.3a	45.3 ± 2.1b

Where: H_2_Ten: (C_13_H_11_N_3_O_4_S_2_); A: (MnC_25_H_27_N_5_O_8_S_2_Cl_2_); B: (CoC_25_H_25_N_5_O_7_S_2_Cl_2_); C: (NiC_29_H_35_N_5_O_13_S_2_); D: (CuC_25_H_27_N_5_O_8_S_2_Cl_2_); E: (ZnC_25_H_23_N_5_O_6_S_2_Cl_2_). Values were recorded as the mean inhibition percentage of fungal growth (three replicates) ± SDs. Values followed by different letters in each vertical column are significantly different according to *Tukey* B test at *P* < 0.05. Cont (PDA): potato dextrose agar (negative control). Azoxy. +ve cont: Positive control Azoxystrobin (0.8 µL/mL).

**Table 6:** IC_50_ of the tested compounds against the three cancer cell lines according to the MTT assay.

Compound Code	IC_50_ (µM) ± SD		
	HCT-116	MCF-7	HepG2
H_2_Ten	16.3 ± 5.1	11.9 ± 4.1	10.5 ± 4.1
Phen	0.6 ± 0.3	0.5 ± 0.2	9.8 ± 3.5
(A)	4.2 ± 1.5	3.0 ± 1.1	11.1 ± 3.9
(B)	4.3 ± 1.6	4.2 ± 1.5	11.4 ± 3.8
(C)	18.5 ± 4.9	11.2 ± 3.5	12.6 ± 4.1
(D)	0.2 ± 0.2	0.3 ± 0.2	6.8 ± 2.5
(E)	19.3 ± 4.3	6.3 ± 2.5	12.0 ± 4.1
Doxorubicin	9.4 ± 3.9	6.7 ± 2.9	10.4 ± 3.6

## Supplementary Materials

The supplementary materials are available online.

## References

[1] Weder J.E., Dillon C.T., Hambley T.W., Kennedy B.J., Lay P.A., Biffin J.R., Regtop H.L., Davies N.M. (2002). Copper complexes of non-steroidal anti-inflammatory drugs: An opportunity yet to be realized. Coord. Chem. Rev..

[2] Gonzalez J.P., Todd P.A. (1987). A Preliminary review of its pharmacodynamic and pharmacokinetic properties and therapeutic efficacy. Drugs.

[3] Atkobar Z., Tuncel M. (1996). The polarographic determination of tenoxicam in the pharmaceutical preparations. Anal. Lett..

[4] Blake D.W., Bjorksten A.R., Libreri F.C. (1997). Oral Tenoxicam for Peripheral Orthopaedic Surgery: A Pharmacokinetic Study. Anaesth. Intens. Care.

[5] Tamasi G., Bernini C., Corbini G., Owens N.F., Messori L., Scaletti F., Massai L., LoGiudice P., Cini R. (2014). Synthesis, spectroscopic and DFT structural characterization of two novel ruthenium(III) oxicam complexes. In vivo evaluation of anti-inflammatory and gastric damaging activities. J. Inorg. Biochem..

[6] Cini R., Tamasi G., Defazio S., Hursthouse M.B. (2007). Unusual coordinating behavior by three non-steroidal anti-inflammatory drugs from the oxicam family towards copper(II). Synthesis, X-ray structure for copper(II)–isoxicam, –meloxicam and –cinnoxicam-derivative complexes, and cytotoxic activity for a copper(II)–piroxicam complex. J. Inorg. Biochem..

[7] Nesseem D.I., Eid S.F., El-Houseny S.S. (2011). Development of novel transdermal self-adhesive films for tenoxicam, an anti-inflammatory drug. Life Sci..

[8] Erbas M., Simsek T., Kiraz H.A., Sahin H., Toman H. (2015). Comparison of the effectivity of oral and intra-articular administration of tenoxicam in patients with knee osteoarthritis. Rev. Bras. Anestesiol..

[9] Baran E.J. (1995). Quimica Bioinorganica.

[10] Adam A.M.A. (2018). Ca(II), Sr(II) and Ba(II) ion interaction with the rheumatoid arthitis drug tenoxicam: Structural, thermal, and biological characterization. Appl. Organometal. Chem..

[11] McCormick D.L., Phillips J.M., Horn T.L., Johnson W.D., Steele V.E., Lubet R.A. (2010). Over expression of Cyclooxygenase-2 in Rat oral cancers and prevention of oral carcinogenesis in rats by selective and non-selective COX inhibitors. Cancer. Prev. Res..

[12] Kumar S.V., Tarun P., Kumar T.A. (2013). Transdermal drug delivery system for non-steroidal anti inflammatory drugs: A review. J. Pharm. Res..

[13] Gurpinar E., Grizzle W.E., Piazza G.A. (2013). COX-independent mechanisms of cancer chemoprevention by anti-inflammatory drugs. Front. Oncol..

[14] Sadeek S.A., Abd El-Hamid S.M. (2016). Synthesis spectroscopic, thermal analysis and in vitro biological properties of some new metal complexes with gemifloxacin and 1,10-phenanthroline. J. Therm. Anal. Calorim..

[15] Kucková L., Jomová K., Švorcová A., Valko M., Segľa P., Moncoľ J., Kožíšek J. (2015). Synthesis, crystal structure, spectroscopic properties and potential biological activities of salicylate‒neocuproine ternary Copper(II) complexes. Molecules.

[16] Zayed M.A., Nour El-Dien F.A., Mohamed G.G., El-Gamel N.E.A. (2004). Structure investigation, spectral, thermal, X-ray and mass characterization of piroxicam and its metal complexes. Spectrochim. Acta A.

[17] Mohamed G.G., El-Gamel N.E.A. (2004). Synthesis, investigation and spectroscopic characterization of piroxicam ternary complexes of Fe(II), Fe(III), Co(II), Ni(II), Cu(II) and Zn(II) with glycine and dl-phenylalanine. Spectrochim. Acta A.

[18] Hay M.T., Hainaut B.J., Geib S.J. (2003). Synthesis and characterization of a novel iron (III) silsesquioxane compound. Inorg. Chem. Commun..

[19] Manonmani J., Thirumuruhan R., Kandaswamy M., Narayanan V., Shanmuga S., Raj S., Ponnuswamy M.N., Shanmugan G., Fun H.K. (2001). Synthesis of copper(II) and nickel(II) complexes using compartmental ligands: X-ray, electrochemical and magnetic studies. Polyhedron.

[20] Cini R., Giorgi G., Cinquantini A., Rossi C., Sabat M. (1990). Metal complexes of the anti-inflammatory drug piroxicam. Inorg. Chem..

[21] Kovala-Demertzi D. (2006). Recent advances on non-steroidal anti-inflammatory drugs, NSAIDs: Organotin complexes of NSAIDs. J. Organomet. Chem..

[22] Galani A., Demertzis M.A., Kubicki M., Kovala-Demertzi D. (2003). Organotin-drug interactions organotin adducts of lornoxicam, synthesis and characterization of first complexes of lornoxicam. Eur. J. Inorg. Chem..

[23] Defazio S., Cini R. (2002). Synthesis, X-ray structure and molecular modelling analysis of cobalt (II), nickel (II), zinc (II) and cadmium (II) complexes of the widely used anti-inflammatory drug meloxicam. J. Chem. Soc. Dalton Trans..

[24] Radecka-Paryzek W., Luks E. (1995). The scandium (III) ion as a template for the synthesis of a hexaaza *Schiff* base macrocyclic ligand. J. Monatsh. Chem..

[25] Costamagna J., Carruso F., Rossi M., Campos M., Canales J., Ramirez J. (2001). Precursors of hexa-azamacrocycles synthesis and X-ray structure of 2,9-diaminophenanthroline-bisacetate-co(ii) and 6,6′-diaminobipyridine-bisacetate-M(ii) (M = Ni, Cu). J. Coord. Chem..

[26] Mohamed A.A., Sadeek S.A., Abd El-Hamid S.M., Zordok W.A., Awad H.M. (2019). Mixed-ligand complexes of tenoxicam drug with some transition metal ions in presence of 2,2/-bipyridine: Synthesis, spectroscopic characterization, thermal analysis, density functional theory and in vitro cytotoxic activity. J. Mol. Struct..

[27] Li Y., Chai Y., Yuan R., Liang W. (2008). Synthesis Spectroscopic and Biological Evaluation of some Levofloxacin Metal Complexes. Russ. J. Inorg. Chem..

[28] Sadeek S.A., Abd El-Hamid S.M. (2016). Preparation, characterization and cytotoxicity studies of some transition metal complexes with ofloxacin and 1,10-phenanthroline mixed ligand. J. Mol. Struct..

[29] El-Tabl A.S., El-Saied A.F., Al-Hakimi A.N. (2007). Synthesis, spectroscopic investigation and biological activity of metal complexes with ONO tri functionalalized hydrazone ligand. Trans. Met. Chem..

[30] Mohamed H.A., Wadood M.A., Farghaly O.A. (2002). Potentiometric and spectrofluorometric studies on complexation of tenoxicam with some metal ions. J. Pharm. Biomed. Anal..

[31] Kose D.A., Kaya A., Necefoglu H. (2007). Synthesis and characterization of bis (N, N-diethylnicotinamide) m-hydroxybenzoate complexes of Co(II), Ni(II), Cu(II) and Zn(II). Russ. J. Coord. Chem..

[32] Lever A.B.P. (1968). The electronic spectra of tetragonal metal complexes analysis and significance. Coord. Chem. Rev..

[33] Mohamed G.G., Abd El-Wahab Z.H. (2003). Salisaldehyde-2-aminobenzimidazole schiff base complexes of Fe(III), Co(II), Ni(II), Cu(II), Zn(II) and Cd(II). J. Therm. Anal..

[34] Masoud M.S., Zaki Z.M. (1988). Synthesis and characterization of 5-(arylazo)thiobarbituric acids and their complexes. Met. Chem..

[35] Macias B., Martinez M., Sanchez A., Dominguez-Gil A.A. (1994). physico-chemical study of the interaction of ciprofloxacin and ofloxacin with polivaientcations. Int. J. Pharm..

[36] El –Gamel N.E.A., Gerlach D. (2008). Uranyl and transition metals chelates of tenoxicam. Crystal structures of trans, trans-[Co(II)-(Hten)_2_(dmso)_2_], trans, trans-[Zn(II)-(Hten)_2_(dmso)_2_] and solvate cis,cis-[UO_2_(VI)(Hten)_2_ (H_2_O)_2_].C2H5OH. Coord. Chem..

[37] Khedr A.M., Draz D.F. (2010). Synthesis, spectroscopic, and thermal analyses of trinuclear Mn(II), Co(II), Ni(II), and Zn(II) complexes with some sulfa derivatives. J. Coord. Chem..

[38] Zordok W.A., Sadeek S.A. (2018). Synthesis spectroscopic characterization, biological studies and DFT calculations on some transition metal complexes of NO donor ligand. J. Mol. Struct..

[39] Ringer A.L., Sherrill C.D., King R.A., Crawford T.D. (2008). Low-lying singlet excited states of isocyanogen. Int. J. Quant. Chem..

[40] Coats A.W., Redfern J.P. (1964). Kinetic parameters from thermogravimetric data. Nature.

[41] Horowitz H.W., Metzger G. (1963). A New Analysis of thermogravimetric traces. Anal. Chem..

[42] El Gammal O.A. (2015). Mononuclear and binuclear complexes derived from hydrazone Schiff base NON donor ligand: Synthesis, structure, theoretical and biological studies. Inorg. Chim. Acta.

[43] Elshafie H.S., Sakr S.H., Sadeek S.A., Camele I. (2019). Biological investigations and spectroscopic studies of new Moxifloxacin/Glycine-Metal complexes. Chem. Biodiver..

[44] Sakr S.H., Elshafie H.S., Camele I., Sadeek S.A. (2018). Synthesis, spectroscopic, and biological studies of mixed ligand complexes of gemifloxacin and glycine with Zn(II), Sn(II), and Ce(III). Molecules.

[45] Patel N.H., Parekh H.M., Patel M.N. (2007). Synthesis, physicochemical characteristics, and biocidal activity of some transition metal mixed-ligand complexes with bidentate (NO and NN) Schiff bases. Pharm. Chem. J..

[46] Zygadlo J.A., Guzman C.A., Grosso N.R. (1994). Antifungal properties of the leaf oils of *Tagetesminuta* L. and *Tagetesfilifolia* Lag. J. Essent. Oil Res..

[47] Elshafie H.S., Mancini E., Camele I., De Martino l., De Feo V. (2015). In vivo antifungal activity of two essential oils from Mediterranean plants against postharvest brown rot disease of peach fruit. Ind. Crop. Prod..

[48] Elshafie H.S., Amato M., De Feo V., Camele I. (2018). Chemical composition and antimicrobial activity of Chia (*Salvia hispanica* l.) essential oil. Eur. Food Res. Technol..

[49] Ceglie F., Elshafie H.S., Verrastro V., Tittarelli F. (2011). Evaluation of olive pomace and green waste composts as peat substitutes for organic tomato seedling production. J. Compost. Sci. Util..

[50] Emam A., Samah N., Loutfy A., Mostafa A.A., Awad H.M., Mohamed M.B. (2017). Cytotoxicity, biocompatibility and cellular response of carbon dots–plasmonic based nano-hybrids for bioimaging. RSC Adv..

[51] Hassan A.S., Mady M.F., Awad H.M., Hafez T.S. (2017). Synthesis and antitumor activity of some new pyrazolo [1, 5-a] pyrimidines. Chin. Chem. Lett..

[52] Flefel E.M., El-Sayed W.A., Mohamed A.M., El-Sofany W.I., Awad H.M. (2017). Synthesis and Anticancer Activity of New 1-Thia-4-azaspiro[4.5]decane, Their Derived Thiazolopyrimidine and 1,3,4-Thiadiazole Thioglycosides. Molecules.

