# Erratum: Elshafie et al. Biological and Spectroscopic Investigations of New Tenoxicam and 1.10-Phenthroline Metal Complexes. *Molecules* 2020, *25*, 1027

**DOI:** 10.3390/molecules26123662

**Published:** 2021-06-16

**Authors:** Hazem S. Elshafie, Sadeek A. Sadeek, Ippolito Camele, Hanem M. Awad, Amira A. Mohamed

**Affiliations:** 1 School of Agricultural, Forestry, Food and Environmental Sciences, University of Basilicata, Viale dell’Ateneo Lucano 10, 85100 Potenza, Italy; hazem.elshafie@unibas.it; 2 Department of Chemistry, Faculty of Science, Zagazig University, Zagazig 44519, Egypt; s_sadeek@zu.edu.eg; 3 Department Tanning Materials and Leather Technology, Centre of Excellence, National Research Centre, El-Buhouth St., Dokki, Cairo 12622, Egypt; hanem_awad@yahoo.com; 4 Department of Basic Science, Zagazig Higher Institute of Engineering and Technology, Zagazig 44519, Egypt; amiraabdeldayem6@gmail.com

The authors of this paper [1] have agreed they would like to add Hanem M. Awad as the fourth co-author, as she made a significant contribution to the cytotoxicity tests.

The authors apologize for not including her in the original paper and for any inconvenience caused to the readers by this change.

The correct authorship should be as follows:

Hazem S. Elshafie ^1^, Sadeek A. Sadeek ^2^, Ippolito Camele ^1,^*, Hanem M. Awad ^3^ and Amira A. Mohamed ^4^

^1^  School of Agricultural, Forestry, Food and Environmental Sciences, University of Basilicata, Viale dell’Ateneo Lucano 10, 85100 Potenza, Italy
^2^  Department of Chemistry, Faculty of Science, Zagazig University, Zagazig 44519, Egypt
^3^  Department Tanning Materials and Leather Technology, Centre of Excellence, National Research Centre, El-Buhouth St., Dokki, Cairo 12622, Egypt
^4^  Department of Basic Science, Zagazig Higher Institute of Engineering and Technology, Zagazig 44519, Egypt
