# Supplementary material for: Biological and Spectroscopic Investigations of New Tenoxicam and 1.10-Phenthroline Metal Complexes
Source: Molecules. 2020 Feb 25;25(5):1027. doi: 10.3390/molecules25051027 (PMC7179110; doi:10.3390/molecules25051027)
Supplement: Supplementary file 1 [file molecules-25-01027-s001.pdf]

Supplementary Materials

Biological and spectroscopic investigations of new tenoxicam and 1.10-phenthroline metal complexes

Hazem S. Elshafie [1], Sadeek A Sadeek [2], Ippolito Camele [1,*], Amira A. Mohamed [3]

[1] School of Agricultural, Forestry, Food and Environmental Sciences, University of Basilicata, Viale dell'Ateneo Lucano 10, 85100 Potenza, Italy; hazem.elshafie@unibas.it (H.S.E.); ippolito.camele@unibas.it (I.C.);

[2] Department of Chemistry, Faculty of Science, Zagazig University, 44519 Zagazig, Egypt; s_sadeek@zu.edu.eg (S.A.S.);
3 Department of Basic Science, Zagazig Higher Institute of Engineering and Technology, Zagazig, Egypt; amiraabdeldayem6@gmail.com (A.A.M.).

* Correspondence: ippolito.camele@unibas.it; Tel.: +39 0971 205544; Fax: +39 0971 205503

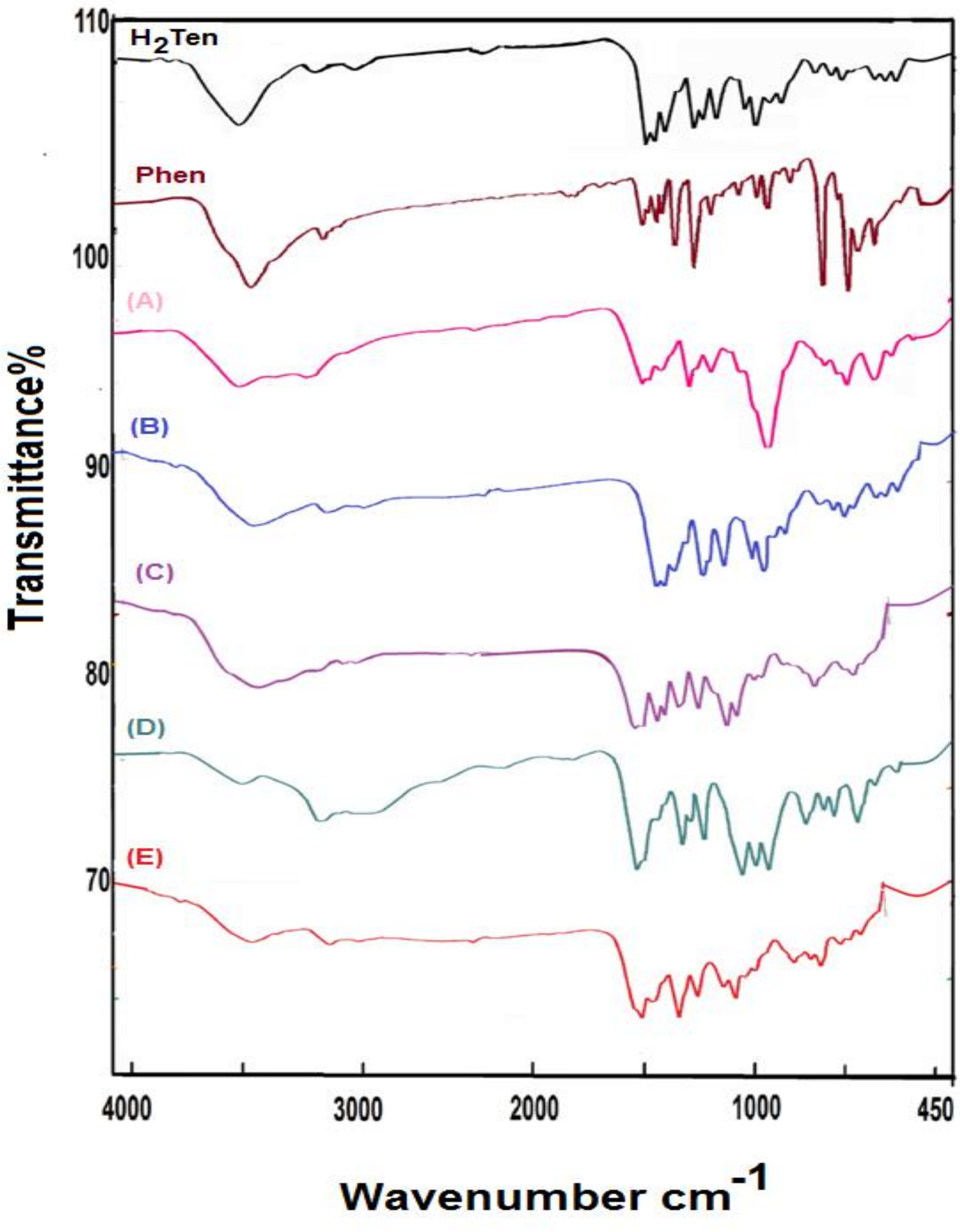

Fig. S1: Infrared spectra for H_2Ten, Phen and their complexes.

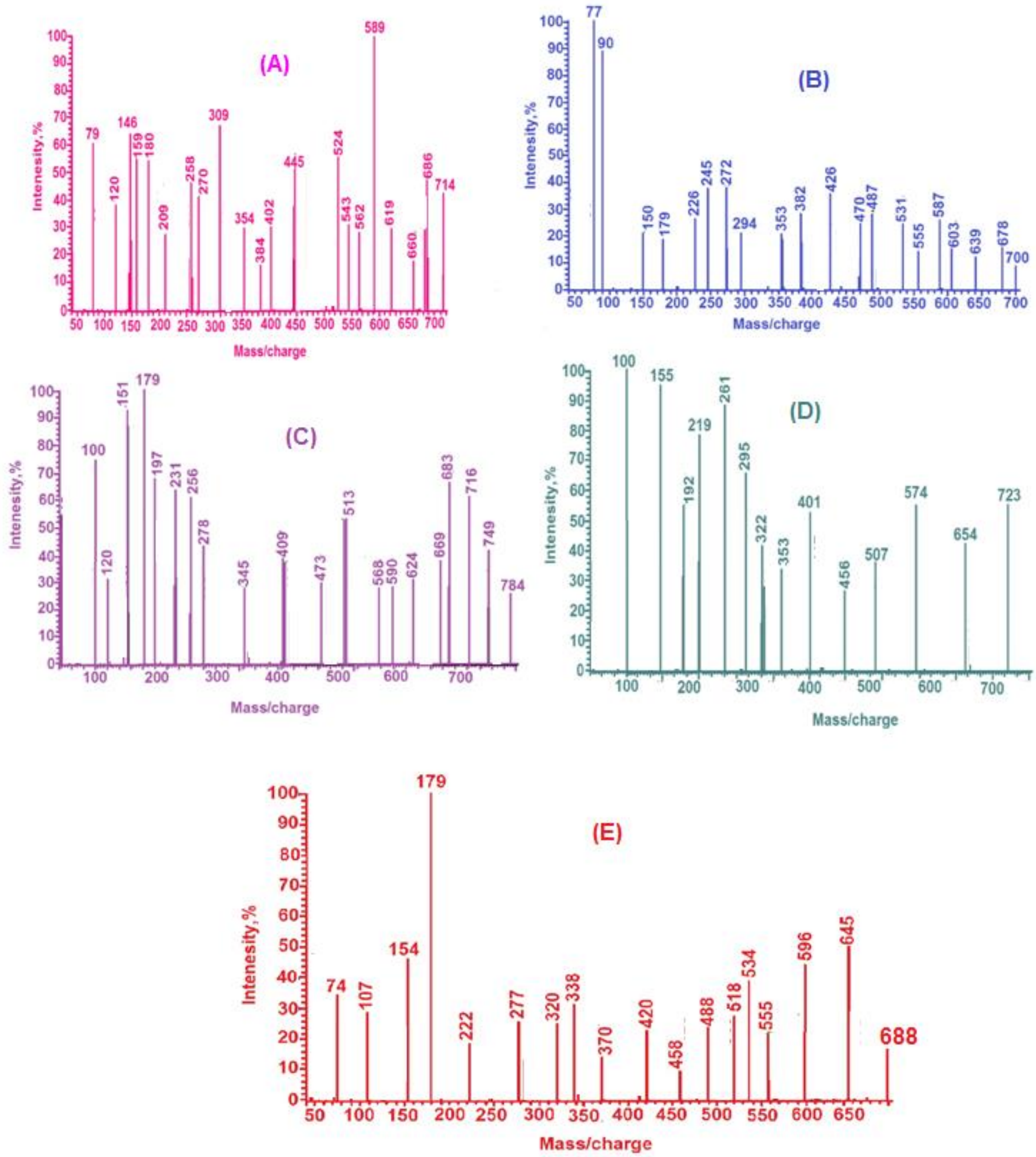

Fig. S2: Mass spectra diagrams for metal complexes

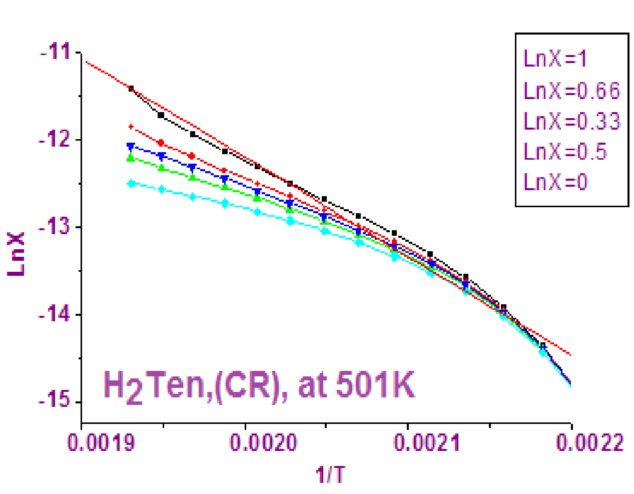
LnX=1
LnX=0.66
LnX=0.33
LnX=0.5
LnX=0
-11
-12
-13
-14
-15
LnX
H_2Ten,(CR), at 501K
0.0019
0.0020
0.0021
0.0022
1/T

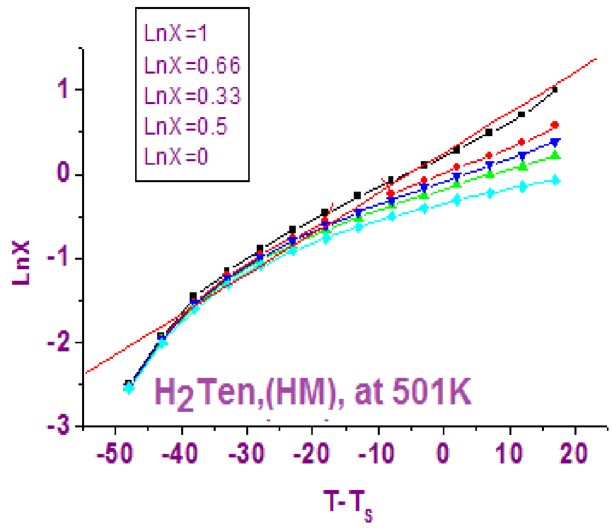
LnX=1
LnX=0.66
LnX=0.33
LnX=0.5
LnX=0
1
0
-1
-2
-3
LnX
H_2Ten,(HM), at 501K
-50
-40
-30
-20
-10
0
10
20
$T-T_s$

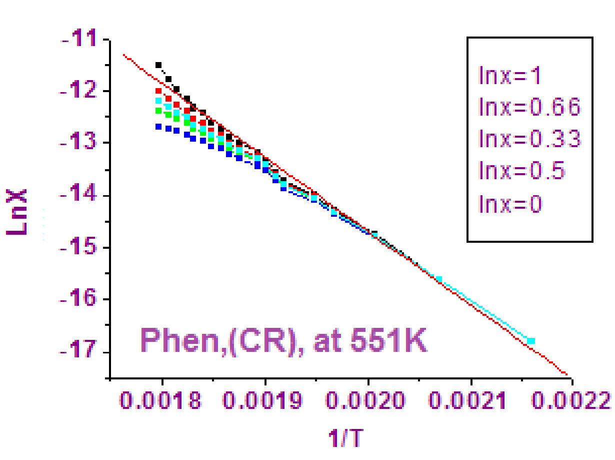
lnx=1
lnx=0.66
lnx=0.33
lnx=0.5
lnx=0
-11
-12
-13
-14
-15
-16
-17
LnX
Phen,(CR), at 551K
0.0018
0.0019
0.0020
0.0021
0.0022
1/T

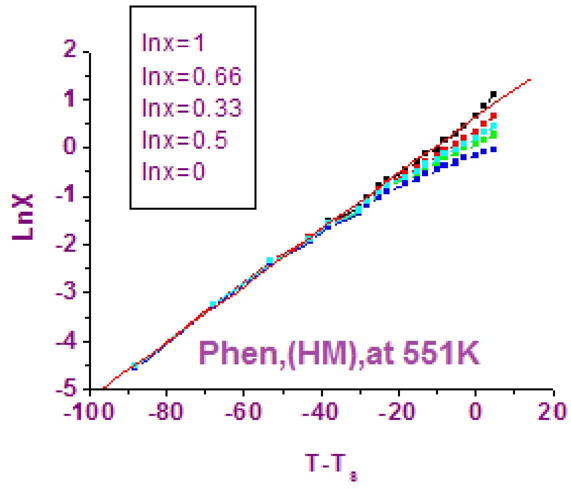
lnx=1
lnx=0.66
lnx=0.33
lnx=0.5
lnx=0
2
1
0
-1
-2
-3
-4
-5
LnX
Phen,(HM),at 551K
-100
-80
-60
-40
-20
0
20
$T-T_s$

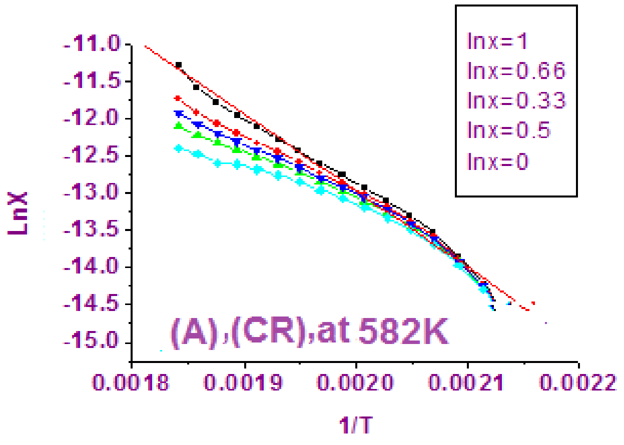
lnx=1
lnx=0.66
lnx=0.33
lnx=0.5
lnx=0
-11.0
-11.5
-12.0
-12.5
-13.0
-13.5
-14.0
-14.5
-15.0
LnX
(A),(CR),at 582K
0.0018
0.0019
0.0020
0.0021
0.0022
1/T

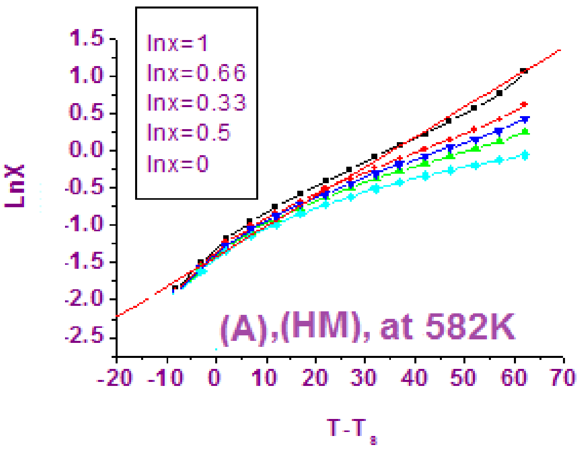
lnx=1
lnx=0.66
lnx=0.33
lnx=0.5
lnx=0
1.5
1.0
0.5
0.0
-0.5
-1.0
-1.5
-2.0
-2.5
LnX
(A),(HM), at 582K
-20
-10
0
10
20
30
40
50
60
70
$T-T_s$

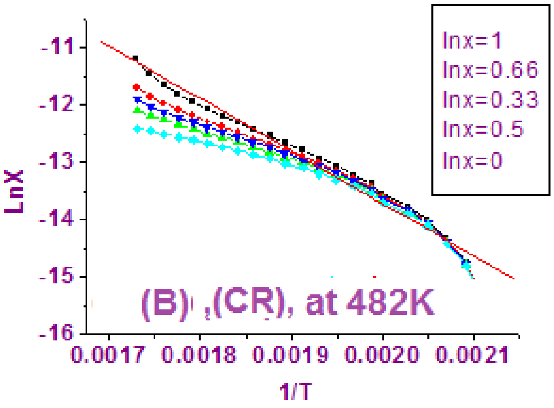
lnx=1
lnx=0.66
lnx=0.33
lnx=0.5
lnx=0
LnX
1/T
(B) ,(CR), at 482K

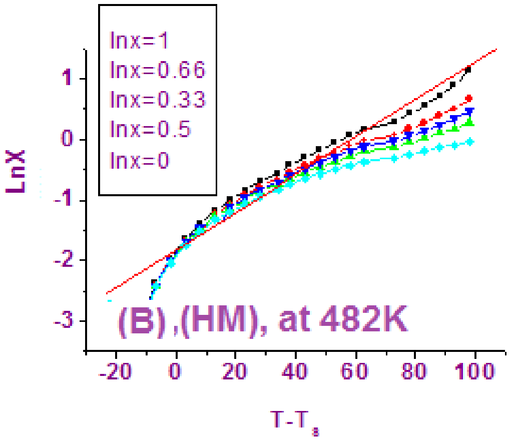
lnx=1
lnx=0.66
lnx=0.33
lnx=0.5
lnx=0
LnX
T-Tg
(B) ,(HM), at 482K

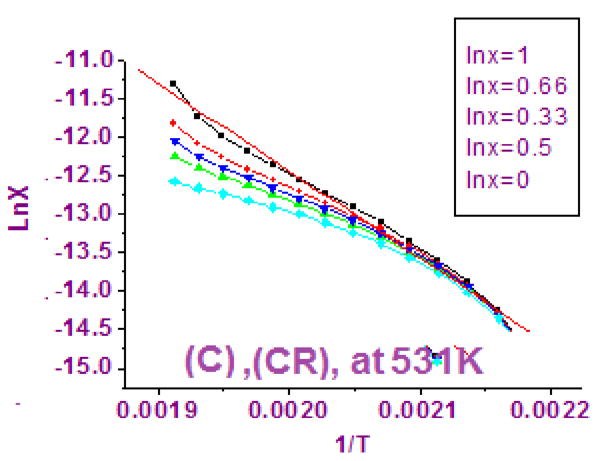
lnx=1
lnx=0.66
lnx=0.33
lnx=0.5
lnx=0
LnX
1/T
(C) ,(CR), at 531K

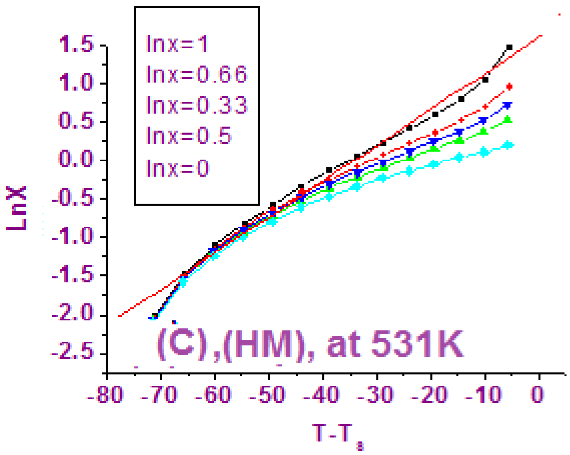
lnx=1
lnx=0.66
lnx=0.33
lnx=0.5
lnx=0
LnX
T-Tg
(C),(HM), at 531K

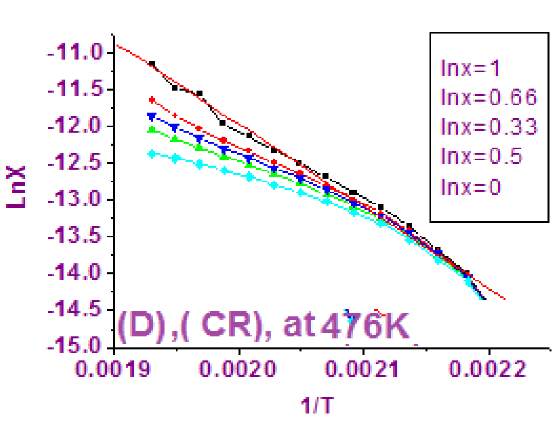
lnx=1
lnx=0.66
lnx=0.33
lnx=0.5
lnx=0
LnX
1/T
(D),(CR), at 476K

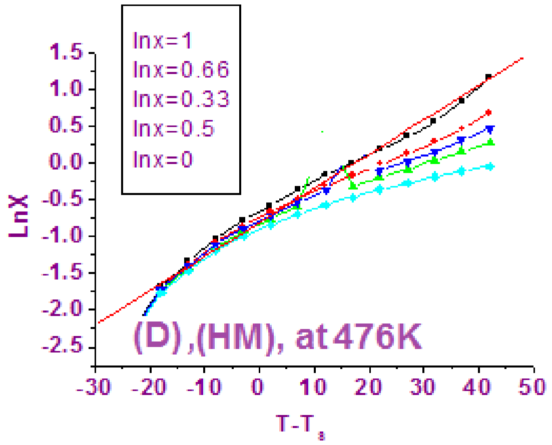
lnx=1
lnx=0.66
lnx=0.33
lnx=0.5
lnx=0
LnX
T-Tg
(D) ,(HM), at 476K

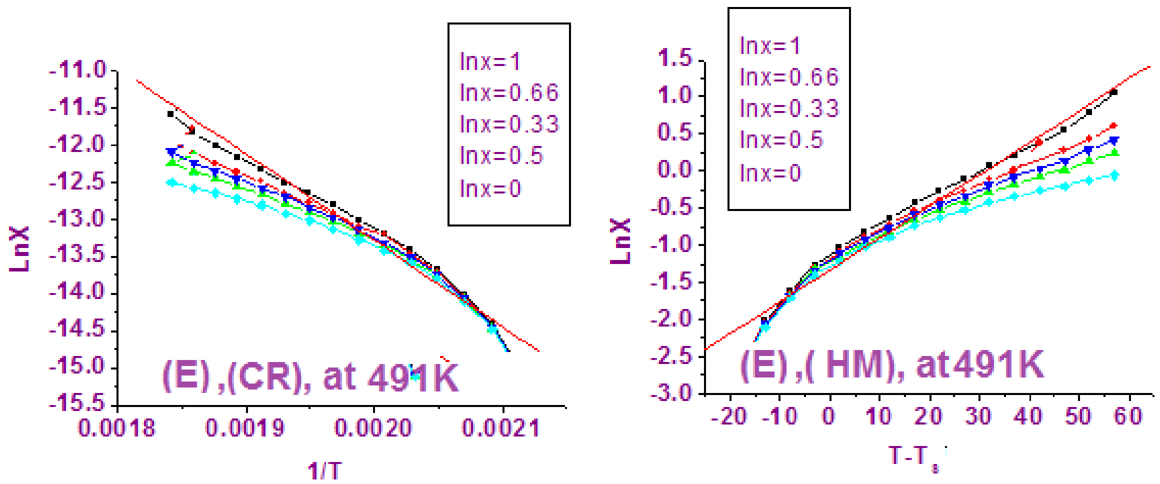

Fig. S3: The diagrams of kinetic parameters of H_2Ten, Phen and their complexes using Coats-Redfern (CR) and Horowitz-Metzger (HM) equations.

Table S1: Elemental analysis and physico-analytical data for H_2Ten, Phen and their complexes

Compounds M.Wt. (M.F.)	Yield%	Mp/°C	Color	Found (Calcd.) (%) C	H	N	M	Cl	μ_{eff}(B.M.)	Λ S cm² mol⁻¹
H_2Ten 337.368($C_{13}H_{11}N_3O_4S_2$)	-	211	Yellow	46.20 (46.24)	3.23 (3.26)	12.41 (12.44)	-	-	Diamagnetic	5.36
Phen 198.2 ($C_{12}H_{10}N_2O$)	-	100	White	72.72 (72.70)	5.09 (5.08)	14.13 (14.12)	-	-	Diamagnetic	5.20
(A) 715.508($MnC_{25}H_{27}N_5O_8S_2Cl_2$)	87.50	180	Pale orange	41.87 (41.92)	3.72 (3.77)	9.71 (9.78)	7.62 (7.67)	9.88 (9.92)	5.44	172.20
(B) 701.508($CoC_{25}H_{25}N_5O_7S_2Cl_2$)	89.00	230	Green	42.70 (42.76)	3.51 (3.56)	9.92 (9.97)	8.35 (8.40)	10.09 (10.12)	5.10	174.00
(C) 784.268 ($NiC_{29}H_{35}N_5O_{13}S_2$)	88.65	220	Pale green	44.32 (44.37)	4.41 (4.46)	8.88 (8.92)	7.42 (7.48)		3.30	157.39
(D) 724.118 ($CuC_{25}H_{27}N_5O_8S_2Cl_2$)	85.00	185	Dark green	41.37 (41.42)	3.68 (4.72)	9.61 (9.66)	8.72 (8.77)	9.72 (9.80)	1.70	171.42
(E) 689.958($ZnC_{25}H_{23}N_5O_6S_2Cl_2$)	84.20	220	Pale yellow	43.42 (43.48)	3.29 (3.33)	10.09 (10.14)	9.42 (9.47)	10.21 (10.29)	Diamagnetic	176.30

Table S2: ^{1}H NMR values (ppm) and tentative assignments for H_2Ten, Phen and their complexes

H_2Ten	Phen	(A)	(B)	(C)	(D)	(E)	Assignments
2.51	-	2.70	2.70	2.48	2.60	2.70	δH, $-CH_3$methyl
-		4.37-4.46	3.42-3.82	3.09-3.72	3.42-3.43	3.43-4.46	δH, H_2O
7.29-8.03	7.26-8.81	7.03-8.36	7.27-8.10	6.82-8.40	7.90-8.02	7.24-8.88	δH, -CH aromatic
8.34		8.74	8.31	8.68	8.05	8.90	δH, -NH amine
13.80		13.77	13.62	13.60	13.56	13.58	δH, OH enolate
